# Supplementary material for: Examining the potential influence of crosslinguistic lexical similarity on word-choice transfer in L2 English
Source: PLoS One. 2023 Feb 1;18(2):e0281137. doi: 10.1371/journal.pone.0281137 (PMC9891524; doi:10.1371/journal.pone.0281137)
Supplement: S1 File — This includes Appendix S1 (Lexical distance), Appendix S2 (Feature edit distance), Appendix S3 (Additional descriptive information), Appendix S4 (Additional technical information), and Appendix S5 (Additional models). (PDF) [file pone.0281137.s001.pdf]

Supporting Information for "Examining the potential influence of crosslinguistic lexical similarity on word-choice transfer in L2 English"

By Itamar Shatz, Theodora Alexopoulou, and Akira Murakami

Published on 2023 in *PLOS One*

TABLE OF CONTENTS

Table of contents ... 1

1 Appendix S1: Lexical distance ... 3

1.1 The term "lexical distance" ... 3

1.2 Validation of Levenshtein distance ... 4

1.3 Limitations of LDN ... 10

1.4 LDN vs. LDND ... 12

2 Appendix S2: Feature edit distance ... 13

2.1 Rationale for feature edit distance ... 13

2.2 Limitations of feature edit distance ... 13

2.3 Our technical approach ... 15

2.4 Descriptive statistics for FDN values ... 18

2.5 FDN-based models ... 23

3 Appendix S3: Additional descriptive information ... 29

3.1 Comparison of our approach with that Rabinovich et al. ... 29

3.2 Comparison of baseline word frequencies ... 30

3.3 Correlations of distance, frequency, and word use ... 30

3.4 Frequency-ratio descriptive statistics ... 37

3.5 Analysis of synonym sets ... 39

4 Appendix S4: Additional technical information ... 42

4.1 Spelling correction ... 42

4.2 Model formula ... 42

4.3 Task random effect....43
4.4 Incidence rate ratio (IRR)....43
4.5 Model diagnostics (assumption checks)....45
4.5.1 Residual plots....45
4.5.1.1 Rationale for diagnostic approach....45
4.5.1.2 Technical details....45
4.5.1.3 Diagnostic plots....48
4.5.1.4 Supplementary models (generalized Poisson)....55
4.5.2 Collinearity....60
4.6 Software used in the analyses....62
5 Appendix S5: Additional models....65
5.1 Models with alternative random effects....65
5.1.1 Random slopes....65
5.1.2 Random intercepts by text....65
5.1.3 Models without the L1 random effect....69
5.2 Baseline models (without distance)....72
5.3 German-only models....77
5.4 Binary-response models....80
5.5 Added-interactions models....84
References....88

1 APPENDIX S1: LEXICAL DISTANCE

1.1 The term "lexical distance"

There is no universal distinction between the terms *lexical distance* and *lexical similarity*, which are often used interchangeably.[1] In the present study, we use *lexical distance* to refer to formal distance between individual L1-L2 words, which are translations of one another. This distance is based on objective phonological distance (specifically, normalized Levenshtein distance—LDN), which serves as a proxy for the subjective similarity between the words that is expected to be perceived by speakers, as supported by the studies outlined in the next sub-section (§1.2).

The reason we use the term lexical *distance* in particular is to distinguish it from other types of language distances, such as morphological distance, in line with prior studies (Bakker et al., 2009; Brown et al., 2008; Gooskens, 2006; Holman et al., 2008b; Schepens et al., 2016; Schepens, van der Slik, et al., 2013b). Here, it is worth noting that lexical distance can serve as a proxy of overall *language distance*, which is sometimes also referred to as *linguistic distance* or *typological distance* (e.g., Ecke, 2015; Llach, 2010),[2] but we do not use it in this sense here, since in our study we only consider the distance of individual L1-L2 word pairs directly, rather than the distance between languages as a whole. Nevertheless, note that we are using a specific type of lexical distance—phonological LDN—as a proxy for overall lexical distance, which can include other factors, such as orthography.[3] In addition, note that other less-common terms are sometimes used for lexical distances that are similar to

[1] Though *increased* distance denotes *decreased* similarity and vice versa, so lexical distance is technically more closely associated with lexical *dissimilarity*.

[2] Though one issue with the term "typological distance" is that it is not always used to refer to overall language distance. Rather, it is sometimes used to refer to distance that is based on grammatical features, such as those that are available in the *World Atlas of Language Structures* (WALS), in order to draw a distinction between it and other types of distance, such as lexical distance that is based on Levenshtein distance in Swadesh lists (Bakker et al., 2009).

[3] Though note that phonological and orthographic similarity tend to be highly correlated. For example, in a recent study on the English and French vocabulary of Dutch speaking children, De Wilde et al. (2021), who also used normalized Levenshtein distance, included only phonological similarity in their analyses, and omitted orthographic similarity, since the two variables were highly correlated and could therefore lead to issues with collinearity. This is also something that these researchers did in another associated study (De Wilde et al., 2020), and is an issue that was raised by other researchers, such as Carrasco-Ortiz et al. (2021), who found a correlation of $r = .782$ between orthographic and phonological distance in their dataset of English and Spanish words. Here too, there were similarly strong correlations between phonological and orthographic distance in the parallel dictionaries, where all the L1s share English's Latin script, both in the case of LDN ($r = .68$, *95% CI* = [.67, .70], $p < .001$), and in the case of LD ($r = .73$, *95% CI* = [.71, .74], $p < .001$). We do not include orthographic distance in our analyses both because of its substantial overlap with phonological similarity in the case of L1s that share English's script, and because we wanted to use consistent analyses for all the L1s in the sample, but orthographic distance is largely meaningless across languages that use different scripts (which includes several of the L1s in the Swadesh-based sample).

the one that we use here, such as *phonological overlap* (Carrasco-Ortiz et al., 2021) or *cognate linguistic distance* (van der Slik, 2010).[4]

Finally, it is important to emphasize that although we use the term "lexical distance" in the present study, our analyses focus on a specific facet of such distance—phonological-based formal distance. However, lexical distance may also encompass other aspects of crosslinguistic similarity, such as semantic and morphological similarity. Nevertheless, given that many studies found a cognate facilitation effect when focusing on formal similarity in a similar way, as shown in the next section, it is unlikely that focusing on such similarity would prevent us from finding a cognate facilitation effect in our own analyses.

1.2 Validation of Levenshtein distance

Here, we outline the extensive use and validation that Levenshtein distance (LD) and its normalized form (LDN) have received in prior research across various fields, including typology, psycholinguistics, and SLA.[5]

First, we open with a notable study by Schepens et al. (2012), which is often cited by other researchers in this context (e.g., Blom et al., 2020; Cenoz et al., 2021; Cop et al., 2017; De Wilde et al., 2020, 2021; Otwinowska & Szewczyk, 2019; Silveira & Leussen, 2015; Wieling et al., 2014). Specifically, in their study, Schepens et al. (2012) conclude the following:

> It is possible to automatically identify large distributions of cognates with respect to form-similarity in various European languages by means of a formalized form-similarity metric such as normalized Levenshtein distance. Applying this metric to a professional translation database, similarity norms were obtained that are comparable to experimentally acquired orthographic similarity ratings (Dijkstra et al., 2010; Tokowicz et al., 2002), and lead to high correlations (around .90) and a large proportion of correctly classified stimuli (over 90%). The obtained distributions were also compared to an account of cross-language similarity based on Gray and Atkinson (r = .72). A common pattern in the degree of orthographic similarity of these

[4] Though the term "cognate lexical distance" is not appropriate to use here, since it refers to the overall distance between languages as calculated based on the proportion of cognates, rather than to distances that are calculated between individual word pairs.
[5] Note that LDN is sometimes also referred to in the literature using similar terms, and especially *NLD* and *nLD*.

distributions was observed within languages of the same family. In our analysis, English showed characteristics of multiple language families (Germanic, Romance). Cognate distributions were computed here using semi-complete lexicons, whereas Gray and Atkinson used only a small set of high frequency words.

In all, our study demonstrated the feasibility and advantages of applying techniques from artificial intelligence to psycholinguistic and linguistic research involving multiple languages. First, the application of the normalized Levenshtein distance function resulted in an automatized selection of more and better stimulus materials for cognate studies on bilingual word processing. Second, the Levenshtein distance function yielded accurate and detailed cross-language similarity distributions for multiple languages, thus allowing a comparison to language family trees. As such, the present study has shown that the Levenshtein distance function can compete with existing similarity measures (such as those proposed by Coltheart, Davelaar, Jonasson & Besner, 1977, and Van Orden, 1987) and can be considered as a new formal and computational model of orthographic similarity, useful for future empirical studies in monolingual and bilingual domains as diverse as those dealing with neighborhood effects, spelling systems, and dyslexia.

(p. 165)

In addition, further support for LD(N) as a measure of lexical distance comes from many other studies.

First, there is substantial support for this measure based on its extensive use in studies pertaining to language classification. For example, in a study that examined lexical distance between 35 Indo-European L1s and Dutch, Schepens et al. (2013b) found a very high correlation (r = .90) between this measure as determined based on the ASJP's Swadesh lists, and distances that are based on shared cognates as determined by Gray and Atkinson (2003) on historical-comparative grounds. Furthermore, Schepens et al. (2013a) found that this measure correlates strongly with crosslinguistic morphological similarity (r = -.65), as determined based on morphological features in the World Atlas of Language Structures. In addition, based on comparisons with other data sources, such as established dialect boundaries, using LD between phonetic strings has been shown to be effective for assessing dialects, for example when it comes to Gaelic (Kessler, 1995) and Dutch (Gooskens & Heeringa, 2004; Nerbonne & Heeringa, 2001). Finally, other studies have found that this

measure leads to accurate language classification as determined based on measures such as expert classification, when it comes to many other languages and dialects (Schepens et al., 2012; Serva & Petroni, 2008; Wichmann et al., 2010).

There is also substantial support for LD(N) based on the high correlation between it and various psycholinguistic measures (Heeringa & Prokić, 2018).[6] For example, Beijering et al. (2008) found a strong correlation between LDN-based distances and intelligibility scores (r = -.86) and perceived linguistic distances (r = .52), in their study of Standard Danish and 17 other Scandinavian language varieties.[7] Similarly, Gooskens (2006) found a correlation of r = -.82 between phonetic LD and intelligibility scores among students from schools in Denmark, Norway, Sweden, and Finland. Furthermore, Gooskens and Heeringa (2004), who examined 15 Norwegian dialects as judged by Norwegian listeners, found a strong correlation between LD and perceptual distance (r = .62 in an experiment where monotonized recordings were used, and r = .67 in an experiment where nonmanipulated recordings were used), leading the researchers to conclude that:

> This shows that dialect distances calculated with Levenshtein distance approximate perceptual distances rather well. We see this as a confirmation of the usefulness of the Levenshtein method, as has been shown before for Dutch dialects. Now we know that the method is also applicable in a language area with a less simple geographic situation than the Dutch one.
>
> (p. 205)

Furthermore, this measure has also been extensively used and validated in the context of second language acquisition (SLA) research, which involved similar analyses as the present study. This includes the following:

- Otwinowska et al. (2020) used LDN to quantify L1-L2 orthographic similarity between words, in their study on the influence of cross-linguistic lexical similarity on the learning of cognates and non-cognates among Polish learners of English. Specifically, they used this measure to show that the cognates and false cognates that they examined were comparable

[6] This is important, since LD/LDN are *objective* measures of language distance, which often serve—including in the present research—as proxies for the *subjective* language distance that learners perceive (i.e., the *psychotypology*), which is the main driver behind the crosslinguistic influence that they experience (Jarvis & Pavlenko, 2008; Kellerman, 1983; Ringbom, 2007; Xia, 2017).

[7] They also found similar correlations when it comes to non-normalized LD (r = -.79 for intelligibility and r = .62 for perceived distance).

in terms of their L1-L2 orthographic similarity, and this measure has been used in similar ways in associated studies (e.g., Marecka et al., 2021; Otwinowska & Szewczyk, 2019).[8]

- Many studies used this measure to assess cognancy. This includes using LD to determine cognancy based on phonological (Sadat et al., 2016) or orthographic transcriptions (Bultena et al., 2020; Y. Zhu & Mok, 2020), using LD to compare cognates and non-cognates based on both phonological and orthographic transcriptions (Carrasco-Ortiz et al., 2021), using LDN to determine cognancy based on orthographic transcriptions (Casaponsa et al., 2015), and using LDN to determine cognancy based on both phonological and orthographic transcriptions (De Wilde et al., 2020).
- In addition, LD/LDN were also used in other studies to assess crosslinguistic similarity of words and its influence on L2 acquisition (De Wilde et al., 2022; van de Ven et al., 2019), to quantify crosslinguistic orthographic overlap of non-identical cognates (Vanlangendonck et al., 2020), and to serve various similar purposes (Cenoz et al., 2021), as have other closely related measures of lexical distance (Dijkstra et al., 2010; Schepens, van der Slik, et al., 2013a).

Note that many of the aforementioned SLA studies also found that this measure of lexical distance is an accurate predictor of various L2 outcomes, including L2 meaning recognition (De Wilde et al., 2022), word processing speed and accuracy (Casaponsa et al., 2015), word recognition (Carrasco-Ortiz et al., 2021), receptive word knowledge (De Wilde et al., 2020), word retrieval (Sadat et al., 2016), translation accuracy (van de Ven et al., 2019), increased errors in the case of gender-incongruent cognates (Bultena et al., 2020), and overall L2 proficiency (Schepens, van der Slik, et al., 2013a).

Finally, in the case of the present study, the classification of L1s based on their lexical distance from English aligns with what we expect based on general language classification. Specifically, based on the distances per L1, which are shown in Table 1, the Germanic and Romance L1s are the lexically closest to English, and all the Indo-European L1s are closer to English than all the non-Indo-Eurpoean L1s (Eberhard et al., 2021).

[8] Otwinowska et al. (2020) also used LDN to quantify orthographic dissimilarity “between a correct L2 translation and a participant’s response that was required to treat the response as correct” (p. 712), and other researchers used this measure for similar comparative purposes (Hanulíková et al., 2012; Marecka et al., 2021).

Table 1. The lexical distances between each L1 and English. This is based on the Swadesh lists, since they contain data for all the L1s in the present sample, and specifically on the data *before* the removal of multi-word entries, unlike the similar table in the body of the paper. The reason for this is that the inclusion of only single-word entries is appropriate for the analyses of individual word pairs, and therefore does not interfere with our main analyses, but could bias comparisons at the language level, where it is important to include all the available word pairs.

			Lexical distance	
L1	Language family [a]	Indo-European [a]	mean	SD
German	Germanic	Y	.656	.27
Italian	Romance	Y	.820	.20
Spanish	Romance	Y	.840	.20
French	Romance	Y	.851	.19
Russian	Slavic	Y	.867	.19
Portuguese	Romance	Y	.878	.18
Japanese	Japonic	N	.892	.15
Arabic	Semitic	N	.912	.12
Mandarin	Sino-Tibetan	N	.920	.12

Note. These values are calculating using English-based tables, where distances are calculated from each English word in the dataset to its closest L1 synonym. It is also possible to calculate these distances using L1-based tables, where distances are calculated from each L1 word to its closest English synonym. However, the distances are quite similar regardless of which option is used (*Spearman's* ρ = 0.97, p < .001); the key differences are that when L1-based tables are used, the Spanish-English distance increases to make it more distant than French, and the Russian-Portuguese distance increases to make it more distant than Portuguese.
[a] Language classifications are based on (Eberhard et al., 2021).

The fact that the Indo-European L1s were found to be lexically closer to English also aligns our expectations based on the measure of linguistic distance proposed by Chiswick and Miller (2005). Specifically, this measure is based on the difficulty that English speakers have acquiring other languages, and has been shown by Chiswick and Miller to predict the difficulty that speakers of those languages will have when acquiring English as an L2. Similarly to our measure of distance, their measure also suggests that all the Indo-European L1s that are included here are closer to English than the non-Indo-Eurpoean L1s.[9]

[9] Their measure ranks languages on a scale of 1–3, where 1 marks the hardest languages to learn (i.e., the most distant) and 3 marks the easiest languages to learn (i.e., the least distant). Out of the L1s included in the present sample, French, Italian, and Portuguese have a ranking of 2.5, German, Spanish, and Russian, have a ranking of 2.25, Arabic and Mandarin have a ranking of 1.5, and Japanese has a ranking of 1. This roughly corresponds to the ranking found here, whereby all the Indo-European L1s are closer to English than the non-Indo-European L1s. The imperfect correlation between their measure and ours is expected, since, as they note, their measure includes various aspects of the language beyond vocabulary, such as syntax.

Furthermore, in this regard, the use of our measure of lexical distance is further supported by Schepens et al. (2013a), who calculated lexical distance in a similar manner as us between 49 L1s and Dutch, and found that increased distance is strongly correlated (r = -.80) with broad L2 proficiency in Dutch.[10] This suggests that distances that are based on this measure strongly predict L2 learnability, in a similar manner as proposed by Chiswick and Miller.

In summary, there is extensive support for our use of LDN as a measure of lexical distance here, including in terms of construct validity. This includes:

- Many studies that validated it by comparing it to other measures of language classification, such as expert cognancy judgments (Brown et al., 2008; Gooskens & Heeringa, 2004; Holman et al., 2008b; Kessler, 1995; Nerbonne & Heeringa, 2001; Schepens et al., 2012; Schepens, van der Slik, et al., 2013b, 2013a; Serva & Petroni, 2008; Wichmann et al., 2010).
- Many studies that validated it by comparing it to psycholinguistic measures, such as perceived distance (Beijering et al., 2008; Gooskens, 2006; Gooskens & Heeringa, 2004; Heeringa & Prokić, 2018).
- Many SLA studies that used it for similar purposes, to assess crosslinguistic similarity (particularly cognancy), and found that it predicts many types of L2 outcomes at the word level (Bultena et al., 2020; Carrasco-Ortiz et al., 2021; Casaponsa et al., 2015; Cenoz et al., 2021; De Wilde et al., 2020, 2022; Hanulíková et al., 2012; Marecka et al., 2021; Otwinowska et al., 2020; Otwinowska & Szewczyk, 2019; Sadat et al., 2016; Schepens, van der Slik, et al., 2013a; van de Ven et al., 2019; Vanlangendonck et al., 2020; Y. Zhu & Mok, 2020).
- The alignment of the overall crosslinguistic lexical distances in our samples with what is expected based on general language classification.

That said, this measure, like all linguistic measures, is imperfect, and we recommend that future work replicate our analyses using other distance measures,[11] as we do ourselves using feature edit distance. Furthermore, it is important to remember that the validation of this measure is itself imperfect, in the sense that the studies that validated it likely had their own

[10] Schepens et al. base this on distances as calculated using Swadesh lists in the ASJP, similarly to us, though they use LDND rather than LDN; this is a closely associated variant of Levenshtein distance, which is discussed in detail in the next sub-section.
[11] For more information on the issues with this measure, see the "Limitations of LDN" sub-section in the paper's methodology. Also, additional criticism of this measure—primarily in the context of language classification—can be found in Greenhill (2011).

limitations and shortcomings, and their methodologies and goals do not always align with our own. Nevertheless, given all the support for this measure outlined above, we believe that its use here is reasonable, and that the outcomes based on it are reasonably reliable and generalizable.

1.3 Limitations of LDN

LDN is limited in several key ways.

First, it treats all character transformations as equal. For example, this means that the English word "fish" /fɪʃ/ has an equal and maximal LDN of 1 from both the corresponding Spanish word ("pez" /pes/) and the Hebrew one ("דג" /dag/), even though the English word is closer phonologically and etymologically to the Spanish word than to the Hebrew one, and could be considered a cognate of the first but not the second.

To partially address this issue, we replicated our analyses using *feature edit distance* (or *phonological edit distance*), and the results of these models replicated our results when using LDN as the measure of distance, as shown and explained in detail in Appendix S2. Briefly, this distance, which has less validation and standardization than LDN, attempts to account for the phonological similarity across segmental units, by assigning different costs to the transformation of different units, based on their phonological features. For example, in the case of "fish" considered above, substituting /ʃ/ with /z/ generally incurs a lower cost than substituting /ʃ/ with /g/, since /ʃ/ and /z/ share more phonological features (e.g., being coronal), so they are more similar to each other from a phonological perspective.

Another limitation of our use of LDN as a measure of lexical distance is that it only looks at one aspect of formal similarity across words (phonological overlap). However, other factors, including both formal ones, such as orthographic depth, and non-formal ones, such as semantic and pragmatic similarities, may also affect crosslinguistic influence. For example, it may be that there is an interaction between orthographic depth and the effects of phonological distance, or that the use of a different script across L1s from different language families moderates the effects of phonological similarity.

Nevertheless, past studies (e.g., Sadat et al., 2016) found a facilitative effect of formal similarity even without considering such factors, as did Rabinovich et al. (2018), who did not investigate the influence of these factors. Furthermore, we addressed this limitation in our

research in two ways. First, as shown in the paper's "Data analysis" section, we used mixed-effects models to control for some of these potential effects through random effects for *word* and *L1*. Second, we replicated our analyses on a sub-sample containing only German speakers (see "German-only models" in Appendix S5), which minimizes some of these issues (e.g., variation in the effects of similarity across language families), and found that these analyses replicated our key findings. However, it will still be beneficial for some future analyses to assess the role of these factors directly.

Finally, note that LDN does not assess *cognancy* directly, which we use in the psycholinguistic sense, of words that have similar meaning and pronunciation/spelling across languages. Rather, it only quantifies the formal similarity between words that are generally similar in terms of meaning. Most notably, this means that there are cases where a large distance does not indicate lack of cognancy, as in the "fish" example above. Nevertheless, as noted in the previous sub-section ("Validation of Levenshtein distance"), LDN is strongly correlated with cognancy (e.g., Schepens et al., 2012), and has been used to estimate cognancy directly in SLA studies that then used it to successfully predict L2 outcomes (Carrasco-Ortiz et al., 2021; Sadat et al., 2016), so we expect to be a reasonable approximation in the context of the present large-scale analyses.[12]

It is important to keep these limitations in mind when interpreting the findings of the study. Nevertheless, as noted in the previous sub-section ("Validation of Levenshtein distance"), this distance has been extensively validated, through research in various fields, such as SLA, psycholinguistic, and language typology. This validation includes, most notably, strong correlations with other measures of distance, such as expert cognancy judgments and perceived language distance (Beijering et al., 2008; Schepens et al., 2012), and the use of this measure in SLA to successfully predict many L2 outcomes at the word level—including in the context of the cognate facilitation effect—such as word recognition and retrieval, in a similar manner as in the present study (Carrasco-Ortiz et al., 2021; Sadat et al., 2016). As such, we believe that the use of LDN is reasonable in the present study. Most importantly, even if it will be unable to perfectly capture *all* of the effects of crosslinguistic similarity, it should be able to successfully capture some of them, as it did in many past SLA studies.

[12] We used the phonological distance directly, rather than using it to estimate cognancy, because there is currently no standardized and well-validated way to determine cognancy based on distance.

1.4 LDN vs. LDND

As noted in the body of the paper, LDN is the normalized version of LD, which accounts for word length by diving the LD between a pair or words by the length of the longer word, to control for variations in word length.

LDN can be further normalized into *LDND*, by dividing it by the mean LDN of all N(N-1)/2 pairings of words with different meanings, to control for shared phonotactic preferences or overlap in phoneme inventories (Bakker et al., 2009, p. 171). However, while the first normalization of LD is usually seen as crucial, the second normalization is controversial and rare (Petroni & Serva, 2010; Wichmann et al., 2010), and none of the SLA or psycholinguistic studies outlined in the previous sub-section (§1.2) used it. Furthermore, the use of LDND can lead to two notable issues. First, it is not sample-independent unlike LDN, so the LDND between two words varies based on which others words from the same languages are included in the analysis, which is not the case for LDN. Second, it minimizes similarity due to shared phonotactic preferences or overlap in phoneme inventories, which *should* be taken into account when assessing lexical distance in the present context, since similarity driven by these causes can influence the perceived similarity of words across languages.

As such, in the present study we use LDN, rather than LDND. Nevertheless, these two measures are generally strongly correlated (Holman et al., 2008a; Pompei et al., 2011; Wichmann et al., 2010), so the impact of using one over the other is likely minor.

2 APPENDIX S2: FEATURE EDIT DISTANCE

2.1 Rationale for feature edit distance

As noted in our discussion of Levenshtein distance in the paper, a notable issue with this measure is that it treats all character transformations as equal, even though this does not accurately represent differences in distances as perceived by learners. For example, this means that the English word "fish" /fɪʃ/ has an equal and maximal LDN of 1 from both the corresponding Spanish word ("pez" /pes/) and the Hebrew one ("דג" /dag/), even though the English word is closer phonologically and etymologically to the Spanish word than to the Hebrew one, and could be considered a cognate of the first but not the second.

A potential way to mitigate this issue is to assign different weights to different character transformations, based on the phonological features of the associated segmental units. The resulting measure, which can be viewed as a modified form of Levenshtein distance, is referred to as *phonological edit distance*, *feature edit distance*, or *feature distance* (FD)(Allen & Becker, 2015; Eden, 2018; Fontan et al., 2016; Hall et al., 2017; Kondrak, 2000; Manurung et al., 2008; McCoy & Frank, 2018; Mortensen et al., 2016; Sanders & Chin, 2009; Schepens, Dijkstra, et al., 2013; L. Zhang, 2018). For example, when using FD, substituting /ʃ/ with /z/ would generally incur a lower penalty than substituting it with /g/, since /ʃ/ and /z/ are share the same value on more phonological features, such as being coronal, so they can be considered more similar to each other from a phonological perspective.

2.2 Limitations of feature edit distance

Though FD might be able to capture phonological similarity more accurate than LD, we decided to use LD(N) as the key measure of similarity in our study, for two main reasons.

First, while there is extensive validation for the use of LD based on research in several fields (as shown in "Validation of Levenshtein distance" in Appendix S1), there is little validation of FD in similar contexts. As such, while LD might potentially be less linguistically motivated than FD, we do know based on prior research that it is able to predict linguistics outcomes fairly well—including when used to predict the influence of crosslinguistic similarity on L2—whereas we do not yet know the same for FD. In fact, the limited research that did investigate the use of FD and similar measures did not find that they

are necessarily better predictors of linguistic outcomes than simple Levenshtein distance (Wieling et al., 2007; Wieling, Nerbonne, et al., 2014). For example, as Wieling et al. (2007, p. 93) state:

> It was found that generally speaking the binary versions approximate perceptual distances better than the feature- based and acoustic-based versions. The fact that segments differ appears to be more important in the perception of speakers than the degree to which segments differ. Therefore we will use the binary version of Levenshtein distance in this article…

Second, the simplicity of LD (compared to FD) presents advantage for replication of analyses, the generalizability of findings, the comparison of findings across studies, and the minimization of researcher degrees of freedom. Specifically, while LD is generally implemented in consistent manner across the various software packages that offer it, which means that calculating LD using different packages/software will lead to the same results, this is not the case of FD, which depends heavily on factors such as:

- Which phonological features are taken into account (Gooskens & Heeringa, 2004; Nerbonne & Heeringa, 1997).[13]
- What weights should be assigned to differences in feature values, and how substitutions should be weighted compared to insertions/deletions.
- Whether different weights should be assigned to different features, and if so, then what weights. This is compounded by the fact that different features could potentially be weighted differently for different populations (e.g., speakers of different L1s, who perceive the different features differently) and in different contexts (e.g., when it comes to assessing perceived distance vs. intelligibility).
- How this distance should be normalized.[14]

[13] For example, in the case of the *PanPhon*, which we use in the present research, /a/ and /æ/ have an FD of 0, since the two segments share identical values for all features in the package's dataset. This can lead to situations where two entries have an FD = 0 but an LD > 0, as in the case of /bambu/ and /bæmbu/ ('bamboo'), which have an FD = 0 but an LD = 1.

[14] Generally, FD is normalized into FDN in a similar manner as LD, though its theoretical maximum is based on the number of segmental units in the longer string, rather than on the number of characters (e.g. /t͡sː/ is viewed as a single segmental unit). However, unlike in the case of LD, where substitutions of non-identical characters always incur a cost of 1, in the case of FD substitutions of non-identical characters generally (depending on the specific type of FD) incur a cost <1, so the theoretical maximum is not actually the length of the longer string, which raises questions regarding what the theoretical maximum should be. For example, should it be based on the maximal number of insertions/deletions together with the maximal possible theoretical substitution given the *global* set of segmental units, or the maximal possible substitution given the *local* set of segmental units? Furthermore, since nearly all substitutions are going to incur a lower cost than the maximum, is it even appropriate to use this maximum in the normalization process?

Furthermore, in this regard, there is also the question of whether to use FD in particular, or a similar measures that attempts to capture crosslinguistic similarities, such as *pointwise mutual information* (PMI)(Wieling, Bloem, et al., 2014) or *naive discriminative learning* (NDL)(Wieling, Nerbonne, et al., 2014).

In summary, although FD might be more linguistically motivated than LD, it is not clear that this is the case and that FD is a better predictor of linguistic outcomes. Furthermore, much methodological work needs to be done on FD to validate and standardize its use, before it can be used with confidence by researchers.

2.3 Our technical approach

We built models that use FD as a predictor, to supplement our main models (which use LD). However, these models, should be interpreted with caution, given the limitations of FD that we discussed above.

To calculate FD for our models, we used *PanPhon*, a Python package that relates IPA segments—both simple (e.g. /t/) and complex (e.g. /t͡sː/)—to their definitions in terms of articulatory features (Mortensen et al., 2016).[15] This includes the following 22 features:

syl [±syllabic]. Is the segment the nucleus of a syllable?

son [±sonorant]. Is the segment produced with a relatively unobstructed vocal tract?

cons [±consonantal]. Is the segment consonantal (not a vowel or glide, or laryngeal consonant)?

cont [±continuant]. Is the segment produced with continuous oral airflow?

delrel [±delayed release]. Is the segment an affricate?

lat [±lateral]. Is the segment produced with a lateral constriction?

nas [±nasal]. Is the segment produced with nasal airflow?

strid [±strident]. Is the segment produced with noisy friction?

[15] The information here is based on version 0.18 of PanPhon. Note that there are also other tools for calculating such distance, such as the *abydos* library in Python (Little, 2018); we chose PanPhon for its features and documentation, but more extensive testing and validation is needed to compare different packages.

voi [±voice]. Are the vocal folds vibrating during the production of the segment?

sg [±spread glottis]. Are the vocal folds abducted during the production of the segment?

cg [±constricted glottis]. Are the vocal folds adducted during the production of the segment?

ant [±anterior]. Is a constriction made in the front of the vocal tract?

cor [±coronal]. Is the tip or blade of the tongue used to make a constriction?

distr [±distributed]. Is a coronal constriction distributed laterally?

lab [±labial]. Does the segment involve constrictions with or of the lips?

hi [±high]. Is the segment produced with the tongue body raised?

lo [±low]. Is the segment produced with the tongue body lowered?

back [±back]. Is the segment produced with the tongue body in a posterior position?

round [±round]. Is the segment produced with the lips rounded?

velaric [±velaric]. Is the segment produced using a velaric airstream mechanism?

tense [±tense]. Is the segment produced with an advanced tongue root?

long [±long]. Does the segment take up two units of length?

This list is based on the information in Mortensen et al. (2016, p. 3478), Mortensen (2015), and Mortensen (personal communication, December 6, 2019)

PanPhon contains the data on these 22 features for different IPA segment, with 3 possible values: in cases where the feature value is specified, it is marked as either + or -, and in cases where it is unspecified, it is marked as 0. For example, table 2 contains the sample values for some of the characters in the PanPhon database.

Table 2. A sample of characters from the PanPhon database, which is used to calculate FD.

ipa	syl	son	cns	cnt	dlr	lat	nas	str	voi	sg	cg	ant	cor	dst	lab	hi	lo	bac	rnd	vel	tns	lng
p	-	-	+	-	-	-	-	0	-	-	-	+	-	0	+	-	-	-	-	-	0	-
z	-	-	+	+	-	-	-	0	+	-	-	+	+	-	-	-	-	-	-	-	0	-
ɲ	-	+	+	-	-	-	+	0	+	-	-	-	-	0	-	+	-	-	-	-	0	-
g	-	-	+	-	-	-	-	0	+	-	-	-	-	0	-	+	-	+	-	-	0	-
gːʲ	-	-	+	-	-	-	-	0	+	-	-	-	-	0	-	+	-	-	-	-	0	+
t͡ʃ	-	-	+	-	+	-	-	0	-	-	-	-	+	+	-	-	-	-	-	-	0	-
u	+	+	-	+	-	-	-	0	+	-	-	0	-	0	+	+	-	+	+	-	+	-
ɑ	+	+	-	+	0	-	-	0	+	-	-	0	-	0	-	-	+	+	-	-	+	-
ɑː	+	+	-	+	0	-	-	0	+	-	-	0	-	0	-	-	+	+	-	-	+	+

Note. The feature values are taken directly from the Nov 11, 2019 release of PanPhon. Some feature names here are trimmed here due to space constraints.

Specifically, we used the *partial_hamming_feature_edit_distance* function,[16] which calculates FD in the following manner:

- An edit that involves an insertion or a deletion incurs a cost of 1.
- An edit that involves going from a certain feature value to an *opposite* feature value incurs a cost of 1/22. For example, if a segment that is [+back] is substituted with a segment that is [-back], a cost of 1/22 is incurred for that particular feature edit.
- An edit that involves going from a *specified* feature value to an *unspecified* feature value and vice versa incurs a cost of 1/44. For example, if a segment that is [+back] is substituted with a segment whose [back] feature is unspecified, a cost of 1/44 is incurred for that particular feature edit.
- An edit that involves going from a certain feature value to an *identical* feature value incurs no cost. For example, if a segment that is [+back] is substituted with a segment that is also [+back], no cost is incurred for that particular feature edit.

The resulting FD was normalized into FDN by dividing it by the length of the longer string in the pair, based on the number of segmental units (e.g., /t͡sː/), since FD focuses on segmental units rather than characters.

Note that whereas LD is standardized, FD is not, as mentioned in the previous section. As such, the FD that we calculated here should be viewed as only one type of FD, and other types of FD are calculated differently and may lead to different outcomes.

2.4 Descriptive statistics for FDN values

There was a moderate-to-strong correlation between FDN and LDN in both the Swadesh lists (r = .40, *95% CI* = [.28, .50], p < .001) and the parallel dictionaries (r = .47, *95% CI* = [.45, .49], p < .001).[17] This suggests that although these two measures have a strong association, as

[16] Alternative functions are available for this purpose in PanPhon. We selected this function because it offered a balance between the two other main functions: *feature_edit_distance*, where insertion/deletions are treated the same as substitutions, and so generally incur a cost <1 (due to the presence of unspecified features), and *hamming_feature_edit_distance*, where transformations from specified feature values to unspecified ones (and vice versa) incur a cost of 1/22, similarly to transformations to opposite feature values. It is not clear that the specific distance that we used is the best one (i.e., the one that best predicts the perceived similarity between words), which highlights the need for validation and standardization of this measure. Nevertheless, this is not crucial for the present research, as the differences between the distances that these measures lead to are small enough that they do not influence our findings.

[17] A similar correlation is found when the full available samples of the Swadesh lists and parallel dictionaries are used (as opposed to the samples that were trimmed for the present study, primarily in terms of focusing only on single-word entries); specifically, this correlation is r = .39 (*95% CI* = [.30, .48], p < .001) in the Swadesh lists,

can be expected, they capture substantially different aspects of crosslinguistic distance, and the use of one rather than the other might influence the results of analyses, at least to some degree.[18]

Figure 1 and Table 3 contain information about the FDN between the L1s in the sample and English. The FDN of all word pairs is available in the data files in the OSF repository (under "Lexical distance & frequency data").

and r = .46 (95% CI = [.44, .48], p < .001) in the parallel dictionaries. In addition, we also calculated some additional correlations that may be of interest to those considering the use of FD(N), though we do not discuss them here further, as they are not too relevant to our analyses. First, LD and FD are more strongly correlated than LDN and FDN (in the Swadesh lists r = .73, *95% CI* = [.68, .78], p < .001; in the parallel dictionaries r = .75, *95% CI* = [.74, .76], p < .001). Second, the correlation between FD and FDN (in the Swadesh lists r = .89, *95% CI* = [.87, .91], p < .001; in the parallel dictionaries r = .82, *95% CI* = [.81, .83], p < .001) is stronger than between LD and LDN, though the correlation between LD and LDN is still substantial (in the Swadesh lists r = .54, *95% CI* = [.46, .61], p < .001; in the parallel dictionaries r = .47, *95% CI* = [.45, .48], p < .001).

[18] Although we do not expect it to change the null findings in the present study, both because past studies found an effect of crosslinguistic similarity while using LD(N), and because the correlation between LDN and FDN means that we would expect to find at least some effect of similarity in our sample, which is not the case.

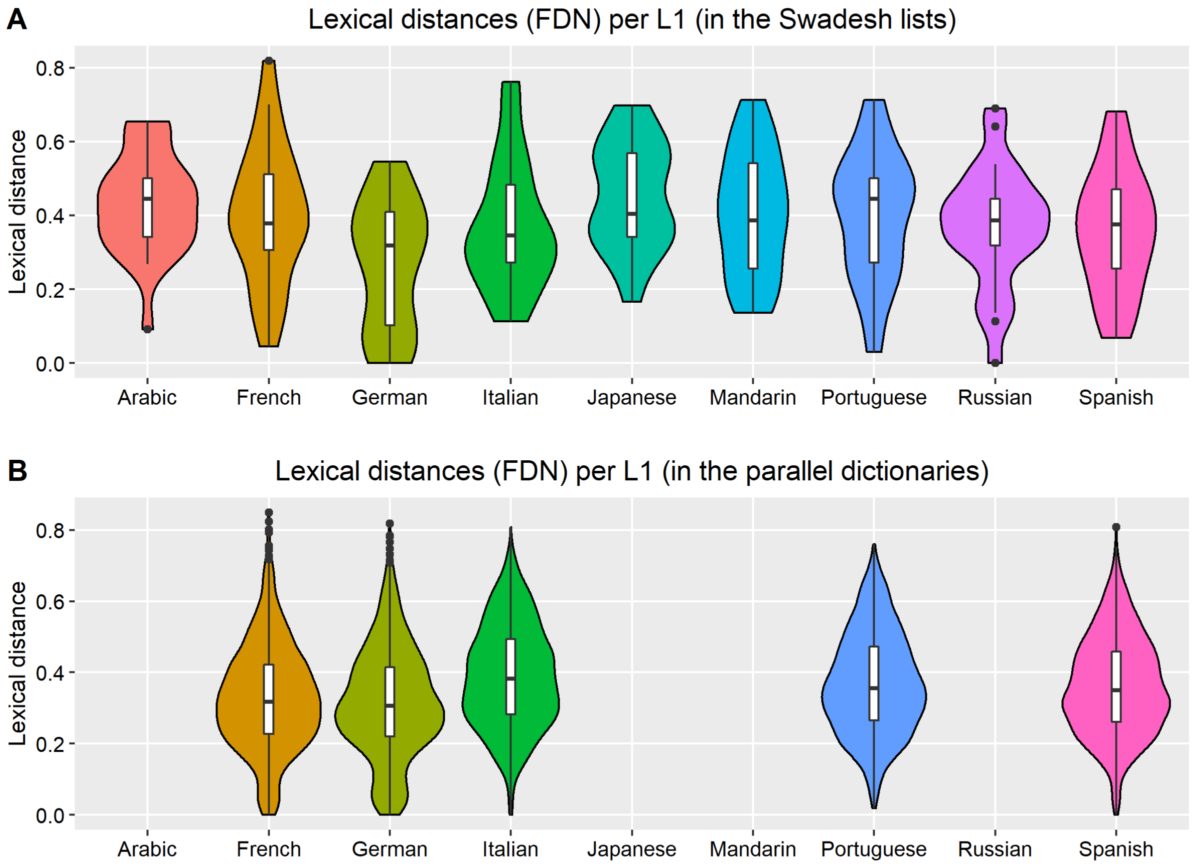

Figure 1. Lexical distance between L1 words English, per L1 in each dataset. The distance is equal to the phonological *FDN* between L1 words and their most lexically similar English counterpart. Within the boxplots, the line inside the box indicates the median, the lower and upper hinges indicate the 1st and 3rd quartiles, the whiskers indicate 1.5 interquartile ranges (IQR) past the hinges, and the dots indicate outliers beyond that. The violin plots indicate an estimate of the probability density of lexical distance for each L1, which can be viewed as the likelihood that a word in each L1 will have a certain lexical distance, where increased width indicates greater likelihood. Data is based on 25 words per L1 in the Swadesh lists and 1,103 words per L1 in the parallel dictionaries (i.e., after the removal of multi-word entries).

Table 3. Statistics about the lexical distances (FDN) between the L1s and English in each dataset. L1s are arranged in order of increasing mean lexical distance in the Swadesh lists.

	Swadesh lists					Parallel dictionaries				
L1	mean	SD	median	IQR	range	mean	SD	median	IQR	range
German	.271	.17	0.32	0.10-0.41	0.00-0.55	.316	.15	0.31	0.22-0.42	0.00-0.82
Spanish	.357	.16	0.38	0.26-0.47	0.07-0.68	.362	.14	0.35	0.26-0.46	0.00-0.81
Russian	.370	.16	0.39	0.32-0.45	0.00-0.69	-	-	-	-	-
Italian	.371	.17	0.35	0.27-0.48	0.11-0.76	.390	.14	0.38	0.28-0.49	0.00-0.81
Portuguese	.400	.17	0.45	0.27-0.50	0.03-0.71	.370	.14	0.36	0.27-0.47	0.02-0.76
French	.404	.19	0.38	0.31-0.51	0.05-0.82	.330	.15	0.32	0.23-0.42	0.00-0.85
Mandarin	.405	.17	0.39	0.26-0.54	0.14-0.71	-	-	-	-	-
Arabic	.432	.14	0.45	0.34-0.50	0.09-0.65	-	-	-	-	-
Japanese	.455	.14	0.40	0.34-0.57	0.17-0.70	-	-	-	-	-

Note. The distance here is the phonological *FDN* from the closest synonym, calculated for the single-word entries in each dataset. There were 225 entries in the Swadesh lists (i.e., rows with an English word and all its corresponding counterparts in a certain L1), with 25 entries for each of the 9 L1s in the dataset. There were 5,515 entries in the parallel dictionaries, with 1,103 for each of the 5 L1s. All counts are after the removal of multi-word entries.

Several key observations can be made about these distances.

First, FDN is much more evenly distributed within each L1 than LDN, primarily due to the lack of ceiling effect present in LDN (i.e., the tendency of words to have the maximal possible LDN of 1). This can likely facilitate analyses using this distance, but it does not necessarily more accurately represent distance between words as perceived by learners.

Second, there are some similarities and differences in the per-L1 differences here compared to those based on LDN, as shown in Table 4 below. Specifically, the similarities are that German is ranked as the closest L1 to English, and that all the Romance L1s (French, Italian, Spanish, and Portuguese) are ranked as closer than all the non-Indo-European L1s (Arabic, Japanese, and Mandarin). The differences are that the ranking is different within the Romance L1, the Indo-European L1s, and the non-Indo-European L1s, and that there are also several differences across these groups, including, most notably, that in FDN Russian is ranked as substantially closer to English than Portuguese and Mandarin, and that French is ranked as being practically as distant from English as Mandarin. These distances are not directly reflective of those between the languages, since they include only single-word entries (as discussed in more detail in the “Validation of Levenshtein distance” section of Appendix S1). Nevertheless, these as shown in the aforementioned section, these distances are expected to be close to the “real” distances between these languages, and as such the results for FDN are highly unexpected, especially in the case of French. This suggests that the present FDN measure is not better than LDN at quantifying crosslingusitic distance.

Table 4. Comparison of the ranking of the L1s based on their distance from English in the Swadesh lists, separately for LDN and FDN.

	LDN		FDN	
Rank	L1	Mean	L1	Mean
1	German	.622	German	.271
2	Italian	.776	Spanish	.357
3	Spanish	.808	Russian	.370
4	French	.813	Italian	.371
5	Portuguese	.848	Portuguese	.400
6	Japanese	.864	French	.404
7	Russian	.881	Mandarin	.405
8	Arabic	.887	Arabic	.432
9	Mandarin	.924	Japanese	.455

2.5 FDN-based models

As with our main models, we used the normalized version of this distance (FDN), which we scaled (by multiplying it by 10) and centered.

We initially built these models using the same fixed and random effects as in our main models. However, the Swadesh-based models in both subcorpora had issues with singular convergence (due to the intercepts and slopes of the L1 random effect), and the parallel-based models did not converge at all.[19]

As such, below (in Tables 5 and 6) we present the results for FDN-based models without the L1 random effect. However, this does not substantially influence our findings, since this effect was very weak in the FDN-based models that contained it and did converge, and the results of the models were functionally identical regardless of the inclusion of this effect, as was the case for the LDN-based models (see the “Models without the L1 random effect” in Appendix S5).

These tables show that the FDN-based models replicate the key findings of the LDN-based models, with a similar null effect of distance and its interaction with proficiency (B = 0.00–0.01, corresponding to IRR = 1.00–1.01), together with strong task effects.

[19] Specifically, they had a “gradient function must return a numeric vector of length 7” error and a “NA/NaN function evaluation” warning.

In addition, we also built FDN-based models using only data from German speakers. This is both to replicate the associated LDN-based models, and because the FDN-based results for the German speakers were consistent with the LDN-based results and with what is expected based on general language classification (as discussed "Validation of Levenshtein distance" in appendix S1), while also being the L1 that is closest to English.

The results of these models are shown in Tables 7 and 8. As with the German-based models that used LDN as the measure of distance, these models replicate the key findings of the main models, in terms of the lack of a substantial effect of distance or of its interaction with proficiency, and in terms of the strong task effects.[20]

Overall, the results from the FDN-based models complement those of the LDN-based main models, and suggest that the null effect in the main models should not be attributed to LDN failing to fully capture the phonological overlap between words, something that is also supported by past validation of Levenshtein distance. However, given the limitations of FD that were above, both in general and within this sample, more work on validating and standardizing FD and similar measures is needed before a conclusive statement can be made on the influence of its use in this context.

[20] The one notable difference is the much weaker effect of frequency here for the parallel-based model in the first corpus, together with an associated increase in the magnitude of the intercept, as is the case with the corresponding LDN-based model. We do not have a clear explanation for this, but it is not crucial for the present analyses, given that the key findings replicate despite of this, and that this was an issue for only one of the four models.

Table 5. Results of the mixed-models with *FDN* as the distance measure, for the Swadesh-based samples. The response variable was the rate of use of the target L2 English words (i.e., their count offset by the total number of words in each text). Under *fixed effects*, *distance* is the phonological FDN between each L2 word and its most lexically similar L1 counterpart (originally 0–1, scaled to 0–10), *proficiency* is the EFCAMDAT L2 proficiency level at which the text was written (1–12, corresponding to CEFR A1–B2), and *frequency* is the baseline Zipf frequency of the target word in English (~1–7.5). Under random effects, τ_{00} represents the SD of the associated random intercepts.

	First subcorpus						Second subcorpus					
Predictor	*B*	SE_B	*IRR*	SE_{IRR}	*z*	*p*	*B*	SE_B	*IRR*	SE_{IRR}	*z*	*p*
(Intercept)	-10.32	0.16	0.00	<0.01	-65.27	<.001	-9.85	0.14	0.00	<0.01	-68.84	<.001
Distance	0.01	<0.01	1.01	<0.01	3.60	<.001	0.00	<0.01	1.00	<0.01	-0.13	.898
Proficiency	-0.04	0.02	0.96	0.02	-2.10	.035	0.00	0.02	1.00	0.02	-0.24	.813
Frequency	3.29	0.21	26.94	5.67	15.65	<.001	3.15	0.19	23.29	4.44	16.52	<.001
Dist:Prof	0.00	<0.01	1.00	<0.01	1.03	.302	0.00	<0.01	1.00	<0.01	1.45	.148
Random effects												
Learner_τ_{00}	0.07						0.24					
Task_τ_{00}	0.40						0.33					
Word_τ_{00}	0.38						0.46					
Task:Word_τ_{00}	1.84						1.36					

Table 6. Results of the mixed-models with *FDN* as the distance measure, for the parallel-based samples. The response variable was the rate of use of the target L2 English words (i.e., their count offset by the total number of words in each text). Under *fixed effects*, *distance* is the phonological FDN between each L2 word and its most lexically similar L1 counterpart (originally 0–1, scaled to 0–10), *proficiency* is the EFCAMDAT L2 proficiency level at which the text was written (1–12, corresponding to CEFR A1–B2), and *frequency* is the baseline Zipf frequency of the target word in English (~1–7.5). Under random effects, τ_{00} represents the SD of the associated random intercepts.

	First subcorpus						Second subcorpus					
Predictor	*B*	SE_B	*IRR*	SE_{IRR}	*z*	*p*	*B*	SE_B	*IRR*	SE_{IRR}	*z*	*p*
(Intercept)	-12.84	0.06	0.00	<0.01	-210.04	<.001	-12.58	0.05	0.00	<0.01	-246.81	<.001
Distance	0.01	<0.01	1.01	<0.01	2.60	.009	0.00	<0.01	1.00	<0.01	0.61	.542
Proficiency	0.12	0.01	1.13	0.01	10.14	<.001	0.04	0.01	1.04	0.01	4.22	<.001
Frequency	2.90	0.06	18.16	1.05	49.95	<.001	2.97	0.05	19.50	0.99	58.51	<.001
Dist:Prof	0.00	<0.01	1.00	<0.01	1.91	.056	0.00	<0.01	1.00	<0.01	0.67	.501
Random effects												
Learner_τ_{00}	0.03						0.05					
Task_τ_{00}	0.03						0.11					
Word_τ_{00}	0.45						0.65					
Task:Word_τ_{00}	2.32						1.50					

Table 7. Results of the mixed-models with *FDN* as the distance measure, for the Swadesh-based samples, using only data from German speakers. The response variable was the rate of use of the target L2 English words (i.e., their count offset by the total number of words in each text). Under *fixed effects*, *distance* is the phonological FDN between each L2 word and its most lexically similar L1 counterpart (originally 0–1, scaled to 0–10), *proficiency* is the EFCAMDAT L2 proficiency level at which the text was written (1–12, corresponding to CEFR A1–B2), and *frequency* is the baseline Zipf frequency of the target word in English (~1–7.5). Under random effects, τ_{00} represents the SD of the associated random intercepts.

	First subcorpus						Second subcorpus					
Predictor	*B*	SE_B	*IRR*	SE_{IRR}	*z*	*p*	*B*	SE_B	*IRR*	SE_{IRR}	*z*	*p*
(Intercept)	-9.81	0.21	0.00	<0.01	-47.29	<.001	-9.35	0.17	0.00	<0.01	-55.11	<.001
Distance	0.06	0.10	1.07	0.11	0.63	.528	0.01	0.08	1.01	0.08	0.15	.884
Proficiency	-0.07	0.02	0.93	0.02	-3.13	.002	-0.01	0.02	0.99	0.02	-0.55	.579
Frequency	2.69	0.24	14.79	3.50	11.38	<.001	2.69	0.19	14.72	2.79	14.19	<.001
Dist:Prof	0.03	0.01	1.03	0.01	2.31	.021	0.00	0.01	1.00	0.01	0.46	.648
Random effects												
Learner_τ_{00}	0.00						0.27					
Task_τ_{00}	0.13						0.20					
Word_τ_{00}	0.21						0.31					
Task:Word_τ_{00}	1.80						1.15					

Table 8. Results of the mixed-models with *FDN* as the distance measure, for the parallel-based samples, using only data from German speakers. The response variable was the rate of use of the target L2 English words (i.e., their count offset by the total number of words in each text). Under *fixed effects*, *distance* is the phonological FDN between each L2 word and its most lexically similar L1 counterpart (originally 0–1, scaled to 0–10), *proficiency* is the EFCAMDAT L2 proficiency level at which the text was written (1–12, corresponding to CEFR A1–B2), and *frequency* is the baseline Zipf frequency of the target word in English (~1–7.5). Under random effects, τ_{00} represents the SD of the associated random intercepts.

	First subcorpus						Second subcorpus					
Predictor	*B*	SE_B	*IRR*	SE_{IRR}	*z*	*p*	*B*	SE_B	*IRR*	SE_{IRR}	*z*	*p*
(Intercept)	-15.47	0.07	0.00	<0.01	-237.29	<.001	-12.46	0.06	0.00	<0.01	-205.98	<.001
Distance	0.00	0.03	1.00	0.03	-0.10	.922	-0.02	0.03	0.98	0.02	-0.99	.322
Proficiency	-0.03	0.01	0.97	0.01	-3.41	.001	0.02	0.01	1.02	0.01	2.77	.006
Frequency	0.11	0.06	1.12	0.07	1.75	.080	2.65	0.06	14.19	0.85	44.37	<.001
Dist:Prof	0.02	<0.01	1.02	<0.01	5.43	<.001	0.01	<0.01	1.01	<0.01	3.85	<.001
Random effects												
Learner_τ_{00}	0.00						0.03					
Task_τ_{00}	0.03						0.03					
Word_τ_{00}	0.35						0.38					
Task:Word_τ_{00}	2.30						1.66					

3 Appendix S3: Additional descriptive information

3.1 Comparison of our approach with that Rabinovich et al.

Our approach differs from that of Rabinovich et al. (2018) in several key ways.

First, in terms of sample, the L2 writings that they examined were produced in a relatively spontaneous setting (on social media), whereas our L2 material was produced in a relatively constrained task-based setting, and within an educational environment. In addition, they examined highly proficient L2 learners, whereas we examine a range of beginner-to-intermediate L2 learners. Also, they covered more L1s in their analyses, but only included Indo-European L1s, whereas we also include a few non-Indo-European L1s (Arabic, Japanese, and Mandarin) in some analyses.

Second, in terms of controlling for background effects, Rabinovich et al. focused on pre-processing the material to minimize background effects (e.g., by randomly shuffling texts by all authors), whereas we control for these effects using mixed-effects models. A notable benefit of our approach is that it allows us to estimate the magnitude of the associated task effects, which are important to SLA research and practices.

Another difference is that Rabinovich et al. analyzed the effects of etymological cognancy as a proxy of formal similarity, whereas we analyze the effects of phonological similarity (a key aspect of formal similarity). While there is a strong association between cognancy and phonological similarity, since cognates are generally more similar phonologically across languages (as discussed in Appendix S1, under "Validation of Levenshtein distance"), these are two different measures, which may lead to different outcomes in certain cases. For example, increased crosslinguistic similarity might facilitate the use of an L2 word even if it this word is not cognate with its L1 equivalent (e.g., if the words share a single but salient consonant due to chance). Also, cognancy involves additional forms of similarity beyond phonological overlap (e.g., pragmatic similarities), which may also influence L2 word choice.

Finally, Rabinovich et al. also examined this effect within synonym sets, whereas we examine this effect in L2 words that are not always parts of synonym sets (as discussed in Appendix S3, under "Analysis of synonym sets"). Furthermore, they focused on synonym sets that contain at least two different etymological paths in particular. A key goal of theirs in doing this was to use L2 word choice to identify speakers' L1, so it made sense for them to focus on a subset of L2 words that are more likely to involve cognate facilitation. Conversely,

our key goal is to understand the influence of learners' L1 on their L2 word choice as it manifests during the SLA process, so we focus on a diverse sample of L2 words, which should be more representative of the words that learners encounter during SLA. Nevertheless, as shown later, our sample contains many crosslinguistically similar L1-L2 word pairs, so we expect to be able to detect associated crosslinguistic influence if it exists in our sample, and this is supported by the precise coefficient estimates that we found for the effects of similarity in our models.

3.2 Comparison of baseline word frequencies

The mean Zipf frequency in the Swadesh lists (N = 25 words per language) was 5.24 (SD = 0.72, median = 5.14, range = 4.15–7.11), and the mean Zipf frequency in the parallel dictionaries (N = 1,103 words per language) was 4.35 (SD = 0.83, median = 4.32, range = 1.87–7.41). Accordingly, the distribution of the Zipf frequencies in our parallel-dictionaries sample—based on the magnitude of the mean, SD, and range—is similar to that of other studies that found a cognate facilitation effect, and our sample also contains substantially more (1,103) words, so this should not be an issue for our analyses.

For example, Carrasco-Ortiz et al. (2021) found a cognate facilitation effect in English-dominant and Spanish-dominant English-Spanish bilinguals when it comes to L2 word recognition. Their sample included 200 word pairs (half of which were cognate across the languages and half of which were not), with a mean Zipf frequency 4.70 (SD = 0.51) for English cognates, 4.64 (SD = 0.52) for English non-cognates, 4.67 (SD = 0.61) for Spanish cognates, and 4.49 (SD = 0.61) for Spanish non-cognates. Similarly, De Wilde et al. (2021) found a cognate facilitation effect in Dutch speakers when it comes to meaning-recognition in L2 French and English. Their sample included 120 L2 English words, with a mean Zipf frequency of 3.98 (SD = 0.64, range = 2.30–5.52), and 104 L2 French words, with a mean Zipf frequency of 3.72 (SD = 0.80, range = 1.70–6.01).

3.3 Correlations of distance, frequency, and word use

Figure 2 contains basic scatterplots with the usage of the target English words in relation to their lexical distance from the corresponding L1 words. These plots show that the datasets contain words with a broad range of lexical distances, and a broad range of rates of usage. In

addition, there appears to be a weak positive association between lexical distance and word usage, since the words with the higher rates of usage are almost exclusively located on the right. This is contrary to the negative correlation that we expect, whereby higher distance is associated with reduced usage. However, this could be due to confounds such as the baseline frequency of the English words, which our mixed-models address.

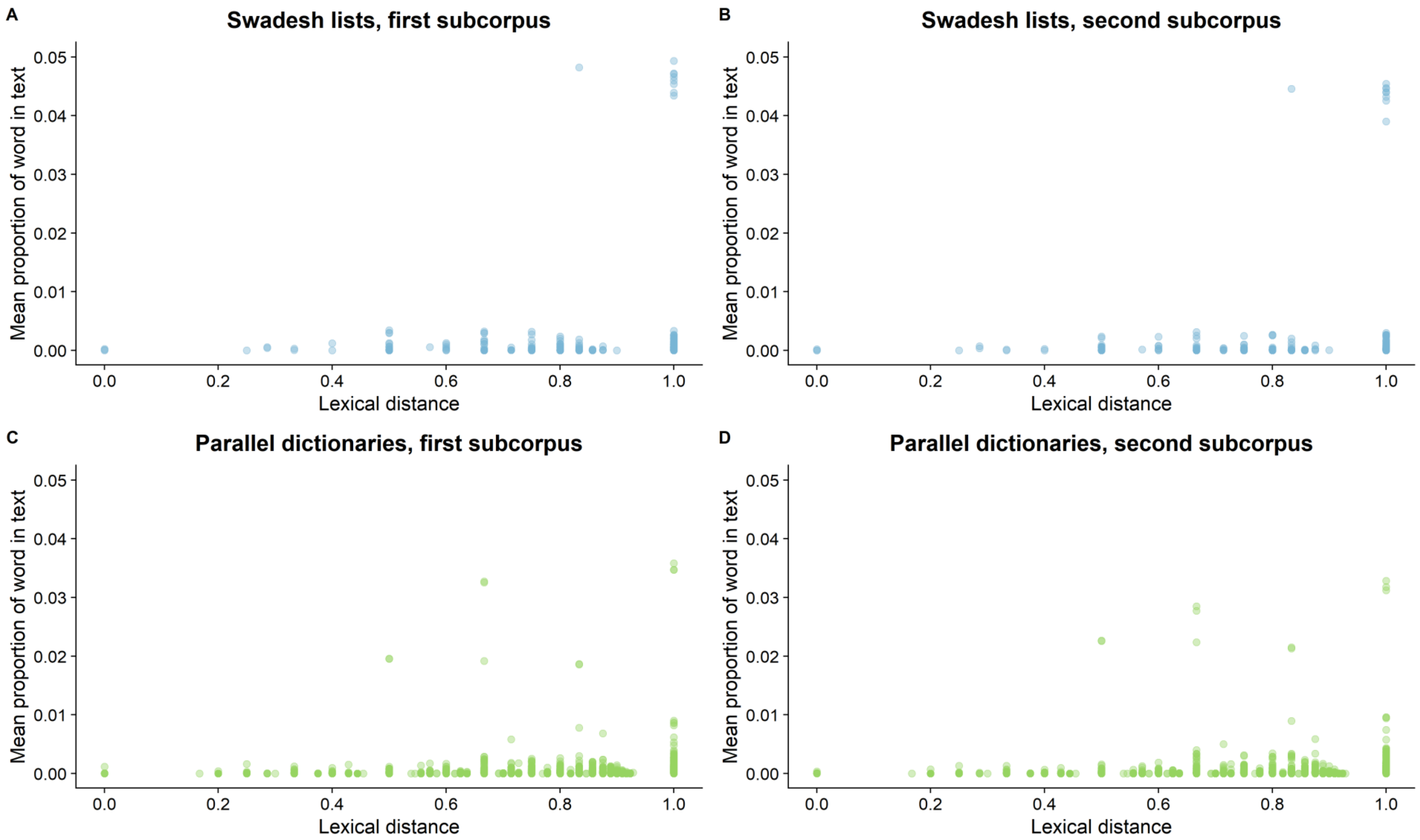

Figure 2. Usage of the target English words, based on their mean proportion in texts (where the proportion of a word in each text is based on the number of times it is used there, divided by the total number of words in the text). Each point is a combination of a target word and a specific L1, since the different L1s can have different distances from English for any given word. Darker shading indicates an overlap in points.

Table 9 shows the raw correlations between lexical distance, baseline frequency of the words in English, and the rate of usage of the L2 English words in the present sample. The Table shows that, in both lexical-distance datasets, there is a significant and substantial positive correlation between the baseline frequency of words and their rate of use in the learner sample, though this correlation is stronger for the words in the Swadesh lists (r = .39–.41) than in the parallel dictionaries (r = .17–.18). In addition, in the Swadesh lists there is also a significant and substantial positive correlation (r = .18) between the lexical distance of words and their frequency, meaning that more distant words are more frequent, but this correlation is not substantial (r = .03) in the parallel dictionaries.

In addition, there is a weak positive correlation between distance and usage for the Swadesh-based samples (r = .10–.11), which might be attributable to the distance-frequency and frequency-usage correlations. This is opposite to the association that we would expect between distance and usage if there was a cognate facilitation effect (assuming no other factors played a role), since *decreased* crosslinguistic distance (i.e., *increased* similarity/cognancy) should lead to *increased* word use. In the case of the parallel dictionaries, there is functionally no correlation between distance and usage (r = .01), which is expected given the almost null correlation between distance and frequency in this dataset, together with the weaker correlation between frequency and word use.

The difference in correlations between the Swadesh lists and the parallel dictionaries can be attributed, in part, to the fact that the parallel dictionaries contain a broader range of words in terms of their baseline English frequencies, including ones that are lower-frequency than in the Swadesh lists (Zipf frequency range of 1.87–7.41 in the parallel dictionaries, compared to 4.15–7.11 in the Swadesh lists). However, as shown in Table 10, when this difference is largely eliminated, by selecting a subset of the parallel dictionaries containing only words with a Zipf frequency of 4.15 and above (as in the Swadesh lists),[21] the *distance-frequency* and *frequency-usage* correlations increase but remain weaker than in the Swadesh lists (respectively, r = .07 and r = .23–.25), and the *distance-usage* correlation remains functionally zero (r = .01 in both subcorpora).

[21] Though there is still a small but significant difference (W = 457718, p < .001) in the mean frequency of words between the datasets, where the mean Zipf frequency in the Swadesh lists is 5.24 (SD = 0.72), and the mean frequency in the parallel dictionaries is 4.91 (SD = 0.54).

Table 9. The raw correlations between lexical distance, frequency, and word usage, presented in the form of *Pearson's r [95% CI] (p)*.

	Swadesh lists		Parallel dictionaries	
	First subcorpus	Second subcorpus	First subcorpus	Second subcorpus
Distance-Frequency [a]	.18 [.05, .30] (.007)		.03 [.01, .06] (.013)	
Frequency-Usage [b]	.39 [.38, .39] (<.001)	.41 [.40, .41] (<.001)	.17 [.16, .17] (<.001)	.18 [.18, .18] (<.001)
Distance-Usage [b]	.10 [.10, .11] (<.001)	.11 [.10, .11] (<.001)	.01 [.01, .01] (<.001)	.01 [.01, .01] (<.001)

[a] The distance-frequency correlation depends only on the source of lexical-distance data (i.e., it is subcorpus-*independent*). N = 225 for the Swadesh lists (based on 25 entries for each of the 9 L1s included there), and N = 5,515 for the parallel dictionaries (based on 1,103 entries for each of the 5 L1s included there).

[a] *Usage* is based on the mean proportion of words in each text (based on the number of times each word is used there, divided by the total number of words in the text). As such, this measure is subcorpus-*dependent*. Sample sizes for it were 212,500 (Swadesh, first), 159,750 (Swadesh, second), 5,235,941 (parallel, first), and 3,915,650 (parallel, second); this is based on the number of lexical-distance entries multiplied by the number of available texts.

Table 10. The raw correlations between lexical distance, frequency, and word usage, presented in the form of *Pearson's r [95% CI] (p)*. Data is based on words in the parallel dictionaries with a Zipf frequency ≥ 4.15 (*n* = 3195, 57.93% of the total words in the parallel dictionaries). For the subcorpus-dependent correlations (i.e., those involving usage), there were 3,033,333 observations in the first subcorpus, and 2,268,450 in the second.

	First subcorpus	Second subcorpus
Distance-Frequency	.07 [.04, .10] (<.001)	
Frequency-Usage	.23 [.22, .23] (<.001)	.25 [.25, .25] (<.001)
Distance-Usage	.01 [.01, .01] (<.001)	.01 [.01, .01] (<.001)

One possibility that was raised, based on the findings of the mixed-models in the paper, is that the cognate facilitation effect does not exist, and was found in other studies due to the confounding influence of factors such as frequency, which we controlled for in the models. While this would be a novel finding in its own right, we do not believe that this is the case.

This is because past studies have found evidence of the cognate facilitation effect even when frequency is controlled for, so we would expect to find this effect here too (Bosma et al., 2019; Carrasco-Ortiz et al., 2021; Casaponsa et al., 2015; Costa et al., 2000; De Wilde et al., 2020, 2022; Hoshino & Kroll, 2008; Otwinowska et al., 2020; Otwinowska & Szewczyk, 2019; Poort & Rodd, 2017; Sadat et al., 2016; Sheng et al., 2016; van de Ven et al., 2019; J. Zhang et al., 2019; Y. Zhu & Mok, 2020). Similarly, in the case of task effects, the aforementioned studies found cognate facilitation using a wide range of methods, including ones where task effects, as conceptualized in the present study, do not play a role, since they were focused primarily on experiment-based investigation of language processing, so it does not appear the us controlling for task effects could explain the lack of cognate facilitation either.

In addition, the correlations that we found here do not lead to a cognate facilitation effect, even without controlling for proper background factors. Specifically, in the case of the Swadesh lists, based on the positive distance-frequency and frequency-usage correlations, we would expect to find an effect *opposite* to cognate facilitation, in the sense that *increased* distance (i.e., *reduced* similarity) will correlate with increased word use, which is in fact what we find for the distance-usage correlation. Furthermore, in the case of the parallel

dictionaries, we would *not* expect to find a similar effect at all, since the correlation between distance and frequency is functionally zero.

Overall, the extensive evidence from past studies shows that the cognate facilitation effect exists even when frequency and other factors are controlled for. Furthermore, the raw correlations between the key variables in our study (lexical distance, baseline frequency, and L2 word usage) show that, when background factors are not properly controlled for, we would expect to find either a null effect or an opposite effect than cognate facilitation. As such, the absence of the cognate facilitation effect in our main models is a novel theoretical finding, that is not merely attributable to the fact that we control for frequency.

3.4 Frequency-ratio descriptive statistics

Table 11 contains descriptive statistics regarding the frequency ratio of the words in the samples, as visualized in Figure 3 of the paper (in the beginning of the Results section). It shows that, on average, target English words were used in equal rates in the sample as in baseline English (i.e., had a frequency ratio near 1). However, all samples contained a range of words with different frequency ratios (total range 0.70–1.58), and this rate was greater in the parallel-based samples, likely due to the inclusion of very low-frequency words. In addition, this inclusion is likely also the reason why more of the words from the parallel dictionaries did not appear in the parallel-based samples at all, as indicated by the substantially higher rate of words with a frequency of 0 in the parallel dictionaries.

Table 11. Descriptive statistics regarding *frequency ratio*, which is the frequency of a word in a given sample divided by its baseline frequency in English. The baseline frequency in English is based on the same frequency measure that we use throughout the paper, as discussed in the "Baseline word frequency" section of the paper. The frequency of use per sample is calculated separately for each combination of a target word and a specific L1, since different L1s can have different distances from English for any given word. The frequencies within the sample are based on 8,500 texts for the Swadesh lists in the first subcorpus, 6,390 for the Swadesh lists in the second subcorpus, 4,747 for the parallel dictionaries in the first subcorpus, and 3,550 for the parallel dictionaries in the second subcorpus. This corresponds to 212,500 observations (number of words per L1 times the number of texts in the sample) for the Swadesh lists for the first subcorpus, 159,750 for the Swadesh lists in the second subcorpus, 5,235,941 for the parallel dictionaries in the first subcorpus, and 3,915,650 for the parallel dictionaries in the second subcorpus.

			Frequency of 0 [b]		Frequency ratio [c]			
Distance dataset	Subcorpus	Words [a]	*n*	*%*	*mean*	*median*	*SD*	*range*
Swadesh lists	first	225	31	13.78	1.03	1.03	0.09	0.70–1.19
Swadesh lists	second	225	31	13.78	1.00	1.00	0.07	0.76–1.19
Parallel dictionaries	first	5,515	2,691	48.79	1.02	1.01	0.12	0.71–1.57
Parallel dictionaries	second	5,515	2,818	51.10	1.01	1.00	0.10	0.70–1.58

[a] *Words* is equal to the number of L1s in the distance dataset (9 in the Swadesh lists, 5 in the parallel dictionaries), times the number of words per L1 (25 in the Swadesh lists, 1,103 in the parallel dictionaries).
[b] Words that did not appear in the sample were assigned a Zipf frequency of 0, in line with Speer (2020), and consequently have a frequency ratio of 0 here. *n* represents the number of such words in the sample, and the *%* represents the percent of such words out of the total words in the sample.
[c] All the *frequency ratio* statistics were calculated while excluding cases with a frequency of zero. A ratio of 1 indicates that a word is used in an equal rate in the sample and in baseline English, whereas a ratio >1 indicates that the word is used more frequently in the sample, and a ratio <1 indicates the opposite.

3.5 Analysis of synonym sets

Rabinovich et al. (2018) use a different approach than us to analyze the cognate facilitation effect, with the key difference being that they examined how cognancy within a set of synonyms corresponding to the same meaning (*synset*) influences word choice, whereas we examined how similarity between words and synonym sets influences word choice. While conducting a similar style of analysis as them is beyond the scope of this study, below we present a brief analysis of our sample in light of the approach used by Rabinovich et al..

Based on our own research and on the conditions in which Rabinovich et al. (2018) found evidence of the cognate facilitation effect, we can identify the following criteria as conditions under which we would expect to find this effect within synonym sets:

- **There must be a communicative need or reasonable opportunity to convey the relevant meaning.** They characterize their sample as involving "spontaneous productions", so in their case it is likely that learners had more opportunities for choosing which meanings to convey than in more constrained task-based settings.
- **The relevant meaning must be able to be conveyed using a synset.** This is because the cognate facilitation effect, as found by them, is based on the contrast in usage between synonyms within a synset.
- **The synonyms must be easily interchangeable.** This is because otherwise, the effects of cognancy may be obscured by other factors that play a role in the choice of specific synonyms out of the synset, and especially frequency effects. In their study, they operationalized this concept by avoiding synsets that were dominated by a single synonym (i.e., where a single synonym accounted for 90% or more of the usage of that synset in their dataset). This means, for example, that a synset such as {*kiss*, *buss*, *osculation*} was excluded, whereas a synset such as {*divide*, *split*} was retained.[22]
- **There must be a mix of cognates and non-cognates in the synset.** Specifically, there must be at least one cognate for the cognate facilitation effect to occur, but there must also

[22] While this is a reasonable operational definition from a practical perspective, especially when working with large-scale datasets, it is important to note that there are various issues with it. For example, some synonyms might not be easily interchangeable due to connotations that they carry, even if they have a similar rate of usage. In addition, the reliance on a strict 90% threshold can lead to issues, such as in a case where a single synonym accounts for 85% of the uses in a corpus, meaning that it is still fairly dominant over the others. Similarly, there can be a difference between a synset with two synonyms that each account for 50% of uses, and a synset with 3 synonyms that has a usage distribution of 50%-49%-1% or 50%-25%-25%. Finally, if a certain L2 word a cognate in many languages, it might become a highly dominant synonym, and therefore be omitted from the sample even though it displays a strong cognate facilitation effect.

be at least one non-cognate against which the cognate stands out.[23] Note that this criterion is L1-dependent, since cognancy of an L2 word is defined based on its relation to an L1 word.

We briefly analyzed our samples to determine to what degree these conditions occur there.

In the Swadesh lists, none of the English words were listed as being a part of a synset. In the parallel dictionaries, out of 1,103 English words that were included in our analyses, 751 (68.09%) were listed as having no synonyms, and 352 (31.91%) were part of a synset. Of those with a synonym, 21 (5.97%) of the entries that originally had two synonyms in the dataset appeared by themselves in the final dataset, due to removal of the other synonym in during the data preparation.[24] Of the 331 entries that were a part of a synset in the present dataset, 304 (91.84%) were part of a synonym pair (i.e., a synset with 2 synonyms), and 27 (8.16%) were a part of a synonym triplet (i.e., a synset with 3 synonyms). As such, there were a total of 161 synsets in our parallel-dictionaries dataset.

When considering how many of these were easily interchangeable, we based our criterion on a similar one as Rabinovich et al., and define an easily interchangeable synset as one where the difference in Zipf frequency between the synonyms is no greater than 1 (i.e., where no synonym is 10 times or more common than the others, since Zipf frequency is on a logarithmic scale). 110 (68.32%) of the synsets (corresponding to 223 entries) fulfilled this criterion, with Zipf frequency differences ranging all the way from 0.00–0.99.

Next, there was the question of which of these synsets contain a difference in lexical similarity that could be characterized as corresponding to cognancy/non-cognancy, since we use a continuous measure of lexical similarity, rather than something that clearly delineates whether a pair of words are cognates or not. As a rough measure, we categorized synset as fulfilling this criterion if at least one of the synonyms had an LDN $\leq .60$ and at least one had an LDN $\geq .80$.[25] Unlike the previous criteria, which were L1-independent, this was L1-dependent, so there were 550 relevant synset combinations (110 synsets for each of the 5 L1s

[23] However, it may also be the case that there can be a facilitative effect of lexical similarity even if there are no cognates in a synonym set, as long as some of the synonyms are substantially more similar to the L1 counterpart than the other synonyms are.

[24] The remaining entries with synonyms did not have any of their synonyms removed during the data-preparation stage.

[25] As with the 90% frequency cutoff proposed by Rabinovich et al., the exact cut-off that was chosen is somewhat arbitrary, and any single cutoff that is used will likely involve a tradeoff between false positives and false negatives. The specific values that were chosen here are based on a manual examination of the data, and while arguments could be made for other values or criteria, it does not appear that this would substantially change the findings of this analysis.

in the parallel dictionaries). Of these, 93 (16.91%), which contain 189 synonyms, fulfilled the cognancy criterion.

Finally, there was the question of whether there was a communicative need for the underlying meanings represented by these synsets. This was determined based on whether at least one of the synonyms in the relevant synsets appeared at least once in a text:

– In the first subcorpus, there were 179,439 rows which represent a combination of one of the above synonyms with a text (while taking learners' L1 into account). Of these, 710 (0.4%) rows had a count > 0 for the target word, meaning that it was used at least once.[26] These represented the use of 63 synsets (67.74% of the original synsets).
– In the second subcorpus, there were 134,190 rows which represent a combination of one of the above synonyms with a text. Of these, 709 (0.53%) rows had a count > 0 for the target word. These represented the use of 64 synsets (68.82% of the original synsets).

Overall, this suggests that, in the present samples, there was a number of cases where words were used from a synset that fulfills the necessary criteria for the cognate facilitation effect (interchangeability, a combination of cognancy/non-cognancy, and a communicative need for the underlying meaning). This aspect of the data should be interpreted with caution, since there are various issues with how these criteria are operationalized and with how synonyms are listed in the datasets in the first place. For example, there are cases where synonyms that fit these criteria are not really interchangeable, as in the case of {*vein*/*artery*}, or are only interchangeable in some situations, as in the case of {*marriage/wedding*}. Furthermore, there is the question of what LDN cutoff should be used to determine cognancy. Nevertheless, even taking such issues into account, it seems that at least some of the entries in the present analyses include cognates as part of a fairly interchangeable synset (e.g., {*woods*/*forest*}, {*stone*/*rock*}, {*carriage*/*wagon*/*cart*}), so it may be possible to use this sample in analyses that are similar to those of Rabinovich et al.

[26] See the paper for more information on this rate of usage.

4 APPENDIX S4: ADDITIONAL TECHNICAL INFORMATION

4.1 Spelling correction

We calculated the counts of words in the datasets using a spelling-corrected version of each text, which comes built-in as part of the EFCAMDAT Cleaned Subcorpus, and which was generated using the *autocorrect* library (McCallum, 2019) in Python, since we are interested in how often learners attempt to use target words, and misspellings could obscure those patterns. Nevertheless, this does not appear to make a practical difference to our analyses, as the correlations between the corrected and uncorrected counts were extremely high (*Pearson's r* = .9954–.9998 for all datasets, with $p < .001$ in all cases, and the *95% CIs* falling no more than .0001 from the estimates. *Spearman's ρ* had similar values, from .9918–.9982, all with $p < .001$).

4.2 Model formula

The formula that we used to build out main models in R was as follows:[27]

```
glmmTMB(
count_target_word ~
ldn_phono_closest_scaled_centered * proficiency_level_centered +
frequency_zipf_centered +
(1 | learner_id) + (1 + ldn_phono_closest_scaled_centered | l1) +
 (1 | task_id) + (1 | target_word) + (1 | task_id:target_word) +
offset(log(wordcount)),
family = poisson,
data = sample_swadesh_first_subcorpus)
```

[27] We made a few minor modifications to the formula's presentation to improve readability. The original formula as used in our analyses is available in the code we provide in the OSF repository (specifically, in the file containing our "main analyses").

4.3 Task random effect

Our models contained a random effect of *task*, to control for all the aspects of each writing task that can influence word choice, such as its prompt, with the exception of the task's associated L2 proficiency level, which we control for using the relevant predictor. This approach accounts for all aspects of task effects in aggregate, and does not disentangle the different aspects.[28] The use of mixed-effects models allows us to assess such task effects despite the fact that each task is associated with only a single proficiency level (Hox et al., 2018; Winter, 2019), and this type of effects structure—where each group in a random grouping variable always takes the same potentially unique value along a continuous predictor—is conventional in both corpus linguistics (e.g., Levshina, 2018) and psycholinguistics (Baayen et al., 2007; Vandenberghe et al., 2021), including in studies on the cognate facilitation effect (e.g., De Wilde et al., 2021).[29]

4.4 Incidence rate ratio (IRR)

As noted in the body of the paper, we exponentiated the coefficient estimates in the mixed-models to derive an *incidence rate ratio* (IRR), in order to facilitate the interpretation of the results, and the *standard errors* (SEs) of the coefficients were then scaled by multiplying them by the exponentiated coefficient estimates (Hox et al., 2018; Sedgwick, 2010).

The IRR itself can be interpreted as the expected change in the rate of the response variable as a factor of a 1-unit increase in the predictor. For example, an IRR of 2 means that a 1-unit increase in the predictor doubles the rate of response (i.e., doubles the rate of use of the target word), while an IRR of 0.5 means that a 1-unit increase in the predictor halves it. An IRR of 1 corresponds to a coefficient estimate (B) of 0, as there is no expected change in the response variable as a result of a change in the predictor.

It is important to note that when combining multiple coefficients, you should *not* add the exponentiated coefficients, but rather multiply them, which is equivalent to exponentiating the added coefficients. For example, consider a situation where you are predicting the IRR of

[28] This operationalization of task is distinct from most notions of *task* within task-based learning and teaching approaches, and we make no claim regarding the impact of any specific aspect of tasks, such as their genre or cognitive complexity (Alexopoulou et al., 2017).

[29] Levshina (2018) used *website* as a random effect, and website *formality* as a predictor. Baayen et al. (2007) used *item* as a random effect, and item *frequency* as a predictor, as did De Wilde et al., (2021). Vandenberghe et al. (2021) used *participant* as a random effect, and participant *vocabulary size* as a predictor.

a word that is 1 unit more frequent than some baseline level, in a learner whose proficiency is 1 unit higher than some baseline level. If the raw coefficient of frequency is 0.5 and that of proficiency is 0.3, then the IRR will be:

$$e^{0.5} \times e^{0.3} = e^{(0.5+0.3)} = 2.2$$

In addition, if you want to predict the IRR of a word that is 1 unit *less* frequent, then you need to take the inverse of the IRR of a word that is 1 unit *more* frequent, since this is equivalent to exponentiating the negative of the associated coefficient. For example, if the coefficient is 0.5, then the IRR of a word that is 1 unit *less* frequent than some baseline level is:

$$e^{-0.5} = \frac{1}{e^{0.5}} = 0.6$$

4.5 Model diagnostics (assumption checks)

4.5.1 Residual plots

4.5.1.1 Rationale for diagnostic approach

When interpreting the diagnostic plots, we follow two notable recommendations from Winter's (2019) relevant work, and namely the focus on visual techniques for diagnostic purposes, and the use of assumption checking on as a way to determine whether there are any major issues with the model. As Winter notes in this regard:

> Newcomers to regression modeling often find it discomforting that the assumptions are assessed visually. In fact, formal tests for checking assumptions do exist, such as the Shapiro-Wilk test of normality. However, applied statisticians generally prefer visual diagnostics (Quinn & Keough, 2002; Faraway, 2005, 2006: 14; Zuur et al., 2009, Zuur, Ieno, & Elphick, 2010). The most important reason for using graphical validation of assumptions is that it tells you more about your model and the data. [Footnote 7: Here are some other reasons: each of these tests also has assumptions (which may or may not be violated), the tests rely on hard cut-offs such as significance tests (even though adherence to assumptions is a graded notion), and the tests may commit Type I errors (false positives) or Type II errors (false negatives)…] For example, the residuals may reveal a hidden nonlinearity, which would suggest adding a nonlinear term to your model (see Chapter 8). Or the residuals may reveal extreme values that are worth inspecting in more detail. One should also remember that a model's adherence to the normality and constant variance assumptions is not a strict either/or. Faraway (2006: 14) says that 'It is virtually impossible to verify that a given model is exactly correct. The purpose of the diagnostics is more to check whether the model is not grossly wrong.'"
>
> (Winter, 2019, pp. 109-110)

The reliance on visual checks is particularly important given the large sample sizes in the present study, which can lead to statistically significant but meaningless deviations from model assumptions (Hartig, 2020).

4.5.1.2 Technical details

All analyses were conducted using R. The models were built using the *glmmTMB* package, which was developed for fitting generalized linear mixed models (GLMMs) (Brooks et al.,

2017).[30] Analysis of residuals for the model diagnostics was performed using the DHARMa package (Hartig, 2021a). This package was chosen as it is dedicated to residual diagnostics for the type of models used in the present study (GLMMs), and it is used in the glmmTMB documentation as the package of choice for this purpose (Bolker, 2020), and it is also widely used by others for this purpose (e.g., Brooks et al., 2019; Gries, 2021).

DHARMa uses an approach to residual diagnostics that addresses common issues with such diagnostics. Full details for the package's approach to diagnostics, and for the rationale behind this approach, can be found in the package's documentation (Hartig, 2021b). However, the key points regarding this approach are the following:

> DHARMa aims at solving these problems by creating readily interpretable residuals for generalized linear (mixed) models that are standardized to values between 0 and 1, and that can be interpreted as intuitively as residuals for the linear model. This is achieved by a simulation-based approach, similar to the Bayesian p-value or the parametric bootstrap, that transforms the residuals to a standardized scale. The basic steps are:
>
> 1. Simulate new data from the fitted model for each observation.
>
> 2. For each observation, calculate the empirical cumulative density function for the simulated observations, which describes the possible values (and their probability) at the predictor combination of the observed value, assuming the fitted model is correct.
>
> 3. The residual is then defined as the value of the empirical density function at the value of the observed data, so a residual of 0 means that all simulated values are larger than the observed value, and a residual of 0.5 means half of the simulated values are larger than the observed value.
>
> …
>
> The key advantage of this definition is that the so-defined residuals always have the same, known distribution, independent of the model that is fit, if the model is correctly specified. To see this, note that, if the observed data was created from the same data-generating process that we simulate from, all values of the cumulative

[30] We chose *glmmTMB* for several reasons, including that it is designed with GLMMs in mind, it supports variants of Poisson models that we used or expected to potentially need (e.g., Conway-Maxwell Poisson), it is substantially faster than many competing packages for the type of models that we built (Brooks et al., 2017), it is well-documented, it interfaces well with other relevant packages (e.g., *broom.mixed*), and it uses a similar syntax as *lme4*.

distribution should appear with equal probability. That means we expect the distribution of the residuals to be flat, regardless of the model structure (Poisson, binomial, random effects and so on).

(Hartig, 2021b)

Specifically, for each model, we ran the four main diagnostic functions that are available in DHARMa. These are explained in detail in the DHARMa documentation (Hartig, 2021b), but we can briefly say the following regarding them and regarding their interpretation:

A. *plotQQunif*- this produces a uniform quantile-quantile plot, to detect deviations from the expected distribution for the model. In a well-specified model, the residuals (black dots) should be plotted over the straight red line.
B. *plotResiduals*- this plots the residuals against rank-transformed predicted values, to detect deviations from uniformity (especially heteroskedasticity or non-linearity).[31] In a well-specified model, the residuals (marked by the shaded grey background and black dots) should be spread homogeneously both vertically and horizontally, and the associated smooth spline (red dashed line) should be plotted over the mean line (solid red line at the horizontal 0.50 mark).[32] Note that, due to the large number of residuals, areas that are *shaded* darker denote a higher concentration of residuals, even if individual black points are not shown there; this is because the function uses a smooth scatterplot instead of a regular scatterplot when the number of residuals is high, to facilitate visual assessment. In addition, stars are used to mark simulation outliers (i.e., data points that are outside the range of simulated values), though it is not a judgment about the magnitude of the residual deviation.
C. *testDispersion*- this tests whether the observed data is more or less dispersed than expected under the fitted model, by comparing the variance of the observed residuals against the variance of the simulated residuals. The key outcome of this test is the ratio between the two, where a ratio < 1 indicates underdispersion, while a ratio > 1 indicates overdispersion.

[31] The predicted values are rank-transformed by default, since this makes patterns easier to spot visually, especially if the distribution of predictors is skewed, as noted in the DHARMa documentation (http://web.archive.org/web/20210803085455/https://rdrr.io/cran/DHARMa/man/plotResiduals.html).

[32] Note that "a scaled residual value of 0.5 means that half of the simulated data are higher than the observed value, and half of them lower. A value of 0.99 would mean that nearly all simulated data are lower than the observed value. The minimum/maximum values for the residuals are 0 and 1." (Hartig, 2021b). Furthermore, due to the way that residuals are transformed in DHARMa, the scaled residuals in a properly fitted model are expected to have a *uniform*—rather than *normal*—distribution.

D. *testZeroInflation*- this compares the observed number of zeros with the zeros expected from simulations. The key outcome of this test is the ratio between the two, where a ratio < 1 indicates that the observed data has fewer zeros than expected, while a ratio > 1 indicates that it has more zeros than expected (i.e., zero-inflation).

The results of the diagnostic tests for each model will be presented in their own figure in the next sub-section, in the form of a panel with 4 tests, each represented by a dedicated plot. Within each figure, plot (A) will correspond to the results from the *plotQQunif* function, plot (B) will correspond to *plotResiduals*, plot (C) will corresponds to *testDispersion*, and plot (D) will correspond to *testZeroInflation*.

Note that, as mentioned in the DHARMa documentation, some minor deviations from perfect patterns (e.g., in the residual plots) can occur due to chance, even in well-specified models. Furthermore, when assessing deviations, it is important to consider the magnitude of the deviation in addition to its significance, as even negligible deviations can be significant in large samples.

4.5.1.3 Diagnostic plots

The diagnostic plots for the Swadesh-lists models appear in Figures 3 and 4. In each figure, (A) contains the QQ plot, (B) contains the residual plot, (C) contains the dispersion test, and (D) contains the zero-inflation test. These diagnostic checks suggest that the models are fairly well-specified, though they have some underdispersion, particularly in the first subcorpus; the potential consequences of this are discussed at the end of this sub-section, after the diagnostic plots for the parallel-dictionaries models.

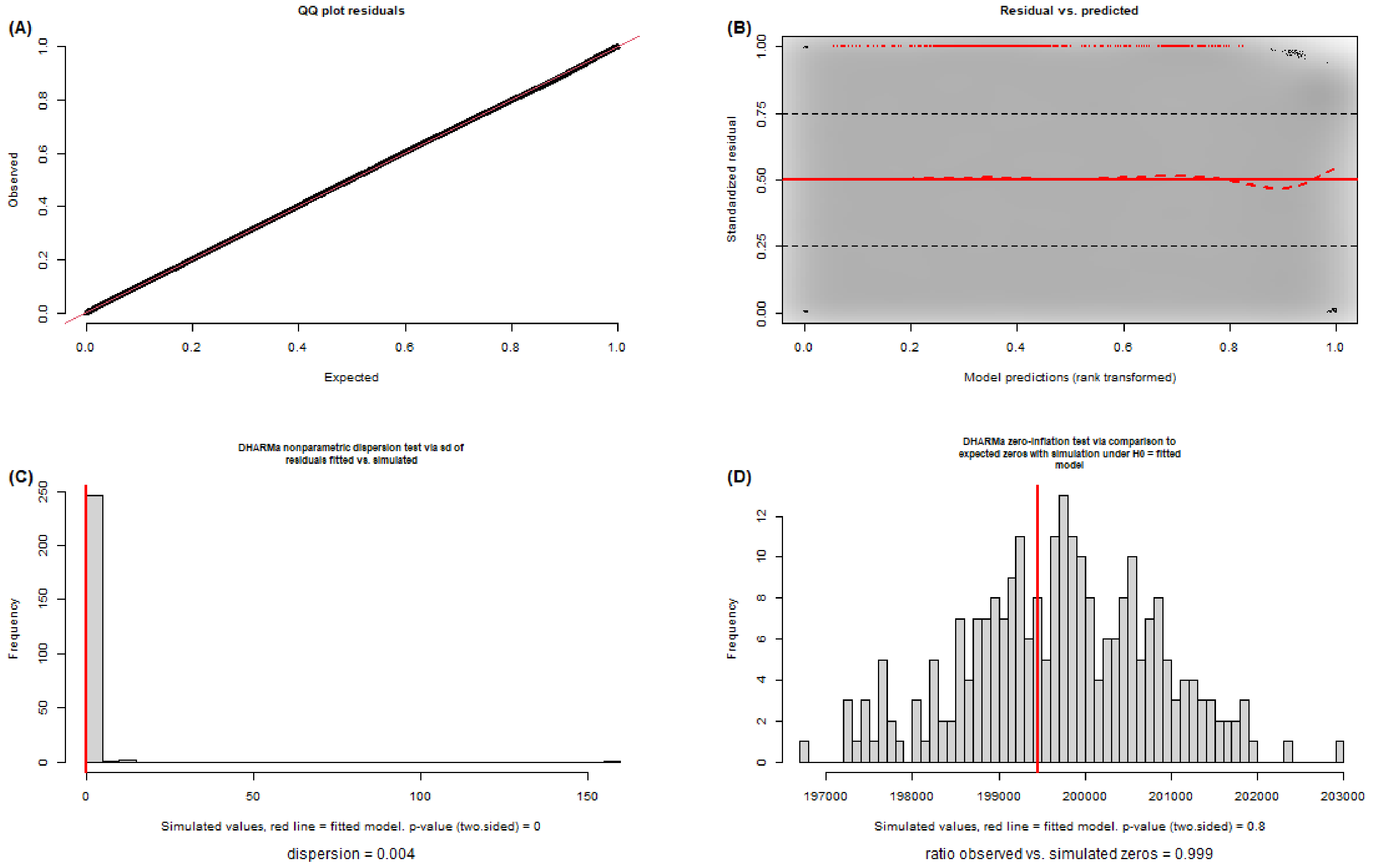

Figure 3. Diagnostics for the Swadesh-lists models (first subcorpus).

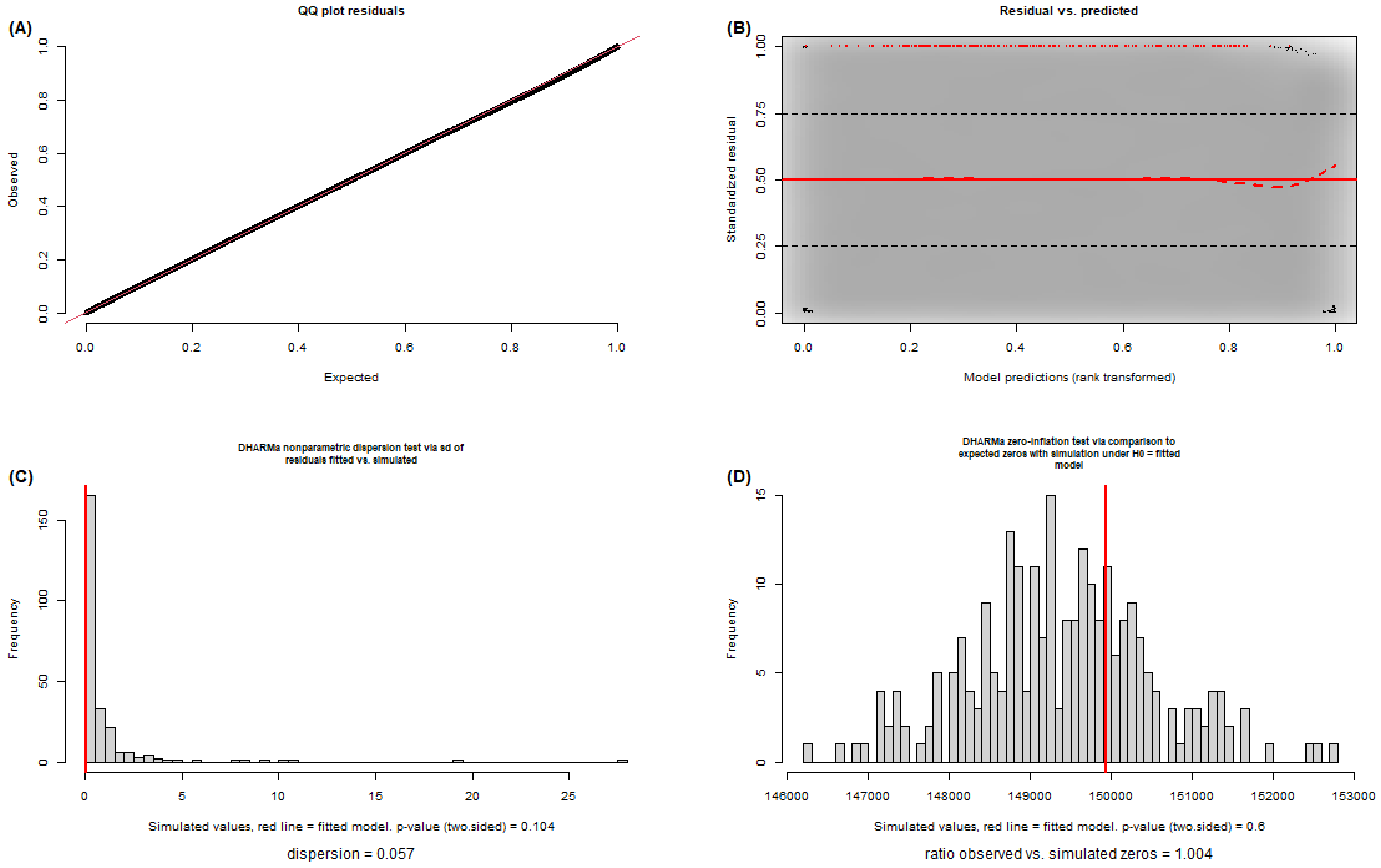

Figure 4. Diagnostics for the Swadesh-lists models (second subcorpus).

In the case of the parallel-dictionaries models, we were unable to run the full diagnostics on the full models, since the large size of the models necessitated memory allocation for the diagnostics that exceeded our available computational resources. To address this, we built new models using sub-samples from the original samples (separately for each subcorpus), containing 2,500,000 randomly selected observations each, and used these for the diagnostics.[33]

The results of these models, which are shown in Table 12, are similar to those of the main models, which supports their use for diagnostic purposes, though the model for the first subcorpus is slightly less well-specified than for the associated main model.[34] The results of the associated diagnostic checks, which appear in Figures 5 and 6, are similar to those of the Swadseh-based models, and suggest that the model are fairly well-specified, though they also have some underdispersion.

[33] The size of 2,500,000 observations was chosen since with a 3,000,000-observations sub-sample we still hit the memory allocation limit for the dispersion and zero-inflation tests.

[34] There are two key differences between the subsample-based model for the first subcorpus and the associated main model. First, this (subsample-based) model had a "singular convergence" warning, likely due to the random intercept for L1 and the associated random slope of distance for L1, though the associated effect sizes were very similar to those in the main models (i.e., functionally 0). Second, the frequency predictor in the subsample model is underestimated, as it has a smaller IRR (and SE) than in the main models, though the frequency predictor is still substantial. It is important to keep these differences in mind when it comes to the diagnostics, but they are nevertheless minor enough that this model is reasonable to use for diagnostic purposes, especially given that it is slightly less well-specified than the main model, which makes using it more conservative. In addition, note that, as expected, the differences between the subsample-based model and the main model generally become smaller as the size of the sub-sample increases, and the residual plots also become even closer to what is expected in a well-specified model. For example, when the sub-sample is increased to 3,000,000 observations, though there is still a "singular convergence" warning, the IRR and SE of frequency both become more similar to those of the associated main model (specifically, the IRR becomes 13.23 and the SE becomes 0.80), and the residual plot become even closer to what is expected for a well-specified model (i.e., the slight uptick at the right side of the plot flattens).

Table 12. Results of the mixed-effects models, for the parallel-based samples, using the 2,500,000-observation subsamples that were selected for diagnostics. The response variable was the rate of use of the target L2 English words (i.e., their count offset by the total number of words in each text). Under *fixed effects*, *distance* is the phonological LDN between each L2 word and its most lexically similar L1 counterpart (originally 0–1, scaled to 0–10), *proficiency* is the EFCAMDAT L2 proficiency level at which the text was written (1–12, corresponding to CEFR A1–B2), and *frequency* is the baseline Zipf frequency of the target word in English (~1–7.5). Under random effects, τ_{00} and τ_{11} respectively represent the SD of the associated random intercepts and slopes, and ρ_{01} represents the correlation between random intercepts and associated random slopes (here, *distance* for *L1*).

	First subcorpus						Second subcorpus					
Predictor	*B*	SE_B	*IRR*	SE_{IRR}	*z*	*p*	*B*	SE_B	*IRR*	SE_{IRR}	*z*	*p*
(Intercept)	-13.83	0.06	0.00	<0.01	-224.94	<.001	-12.52	0.05	0.00	<0.01	-236.96	<.001
Distance	0.01	<0.01	1.01	<0.01	2.54	.011	0.00	0.01	1.00	0.01	0.16	.871
Proficiency	0.02	0.01	1.02	0.01	2.16	.031	0.04	0.01	1.04	0.01	4.31	<.001
Frequency	2.21	0.06	9.15	0.57	35.60	<.001	2.91	0.05	18.34	0.96	55.74	<.001
Dist:Prof	0.00	<0.01	1.00	<0.01	0.61	.541	0.00	<0.01	1.00	<0.01	1.58	.115
Random effects												
Learner_τ_{00}	0.03						0.05					
Task_τ_{00}	0.03						0.08					
Word_τ_{00}	0.27						0.58					
Task:Word_τ_{00}	2.47						1.52					
L1_τ_{00}	0.00						0.00					
L1.Distance_τ_{11}	0.00						0.01					
L1_ρ_{01}	1.00						0.87					

Note. L1_ρ_{01} = 1 in the first model due to the “singular convergence” issue discussed earlier. This is not an issue in the corresponding main model, which has more data.

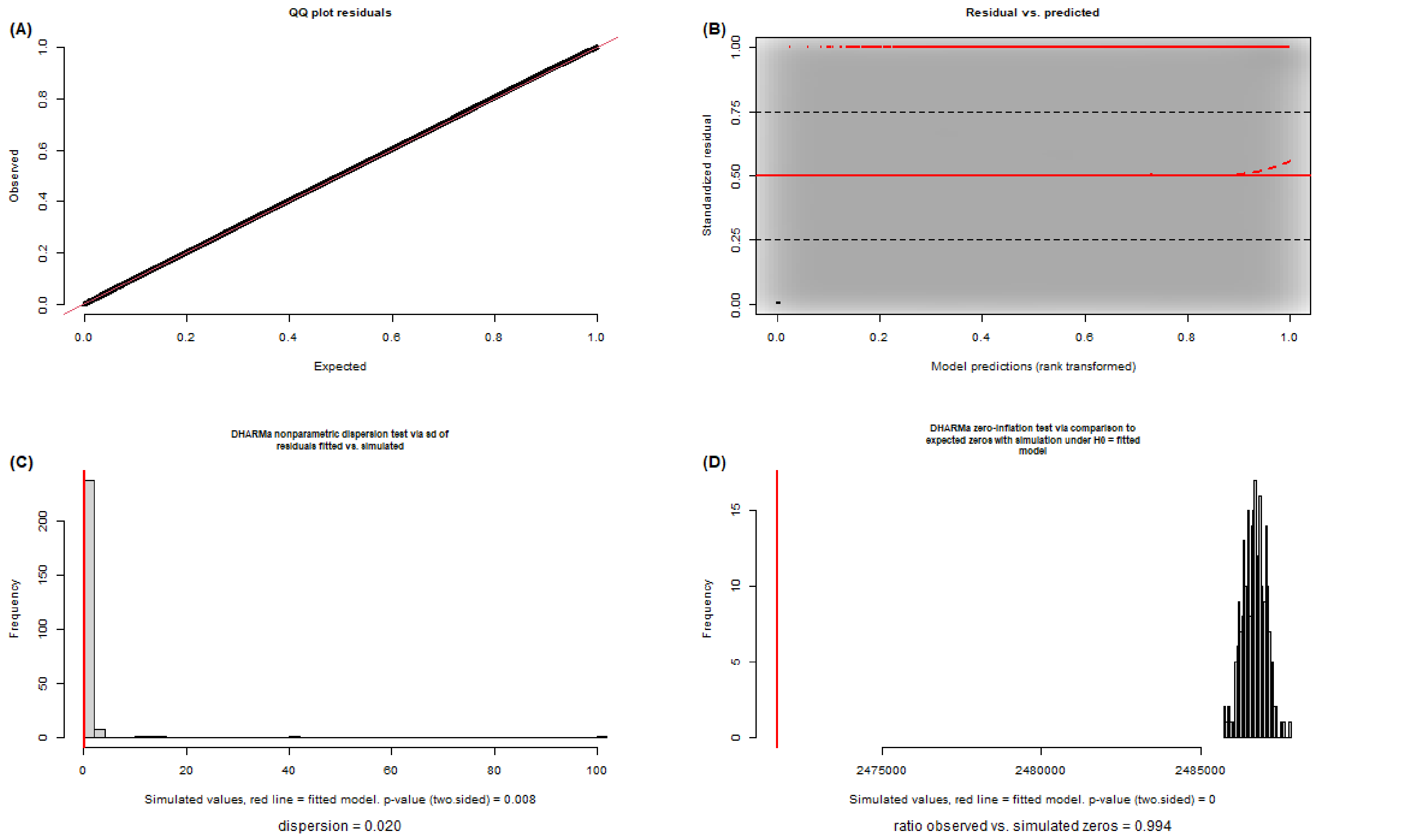

Figure 5. Diagnostics for the parallel-dictionaries models (first subcorpus). The zero-inflation test (5D) looks different here than in the other models, because it involves both a larger sample and a ratio of observed vs. simulated zeros that is slightly farther from 1, so overall there is a larger difference between the observed vs. simulated zeros. This shows there is no zero-inflation here, since there are fewer observed zeros than expected (the ratio of observed vs. simulated zeros is <1), and the ratio is close enough to 1 (0.994) that this is not an issue for the model.

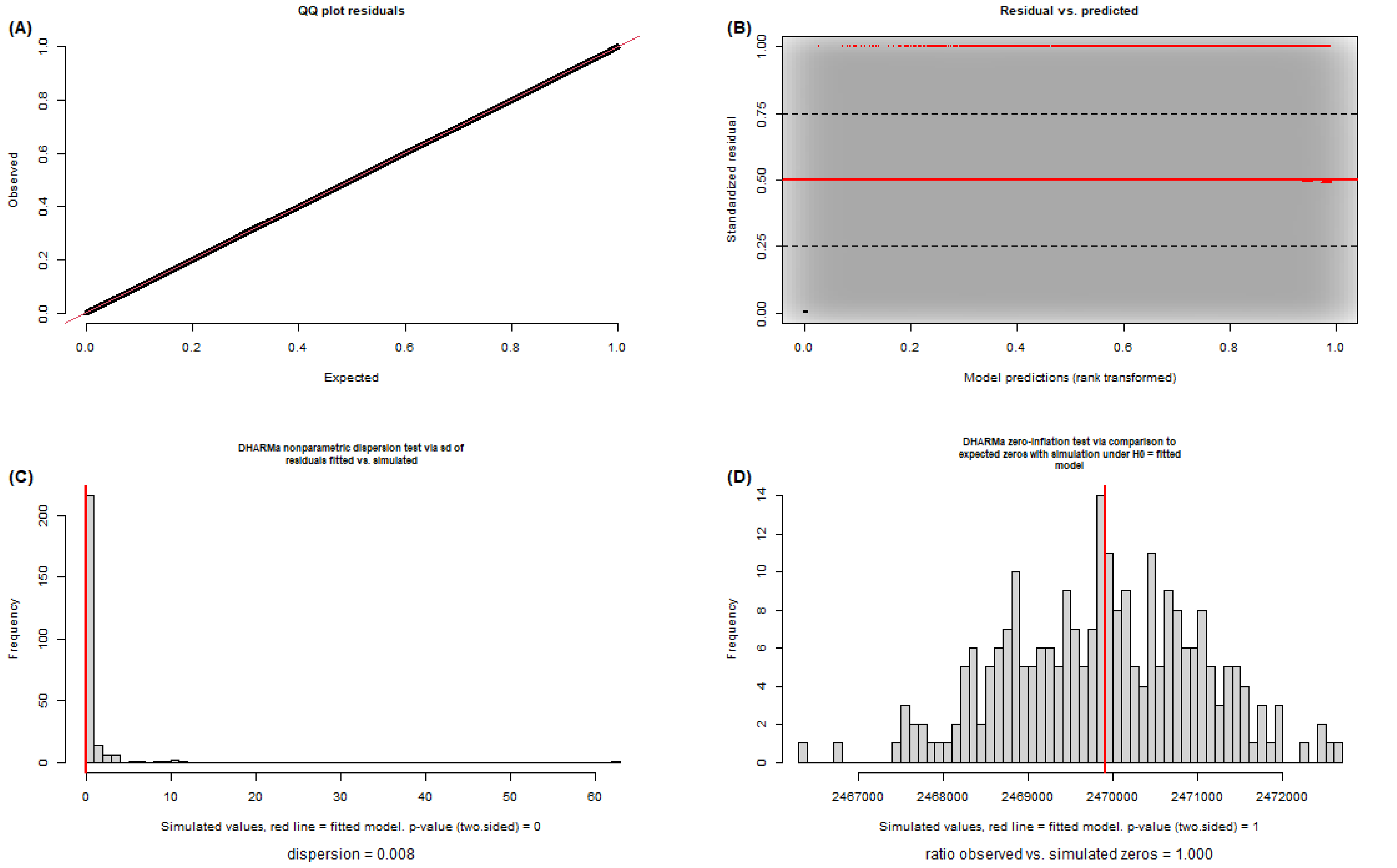

Figure 6. Diagnostics for the parallel-dictionaries models (second subcorpus).

Overall, the diagnostics for the models suggests that the models are fairly well-specified. Specifically, as shown in the graphs in these section—an explanation for which can be found in the previous section (§4.5.1.2) and in Hartig (2021a)—there do not appear to be substantial deviations from the expected distribution from the model (including no zero-inflation), or any substantial deviations from uniformity (i.e., there does not appear to be heteroskedasticity or non-linearity).[35]

The diagnostics do show that some of the models have some underdispersion, which can lead to overestimated SEs, and consequently to overestimated p-values (Brooks et al., 2017, 2019; Dean & Lundy, 2016; Forthmann & Doebler, 2021; Harris et al., 2012; Hartig, 2021b; Sellers & Morris, 2017). However, this underdispersion does *not* invalidate the present findings, given the robust effect sizes that were found across all samples (IRRs very close to 1, with SEs $\leq$ 0.01), since even if these SEs are overestimated, the key patterns of results are still the same, in terms of the lack of effect of distance and of its interaction with proficiency. Essentially, even if these SEs should be smaller than they are, this would only reinforce our certainty regarding the estimated IRRs, and show that they are functionally equivalent to 1, which corresponds to a coefficient estimate of 0 and means that there is no effect. This is further supported by the supplementary models in the next section, which replicate our findings while accounting for underdispersion. In sum, these diagnostics suggest that these models are fairly well-specified, and that they allow us to reliably answer our key research questions.

4.5.1.4 Supplementary models (generalized Poisson)

To account for any underdispersion in the main models, we built supplementary *generalized Poisson* models, which can handle both underdispersion and overdispersion (Brooks et al., 2019; Harris et al., 2012; Sellers & Morris, 2017; F. Zhu, 2012).[36] As shown below, these

[35] Also, note that many past studies on the facilitative effect of crosslinguistic similarity found this effect using similar linear models (e.g., Casaponsa et al., 2015; De Wilde et al., 2020, 2021; Sadat et al., 2016), so we would expect our own linear models to capture such an effect too.

[36] In addition, we also attempted to build *Conway-Maxwell-Poisson* models, which can also handle both underdispersion and overdispersion (Brooks et al., 2017, 2019; Forthmann & Doebler, 2021; Lynch et al., 2014; Sellers & Morris, 2017). The reason for this attempt was that these models might be less prone to convergence problems, though they are also much more computationally intensive (Brooks et al., 2019). Unfortunately, they also had convergence warnings for the Swadesh-based model in the second subcorpus, similarly to the generalized Poisson models, so they were not helpful in this regard, and furthermore, due to their high computational costs, we were unable to get them to converge for the parallel-based samples. Nevertheless, this is not crucial, as the results for these models in the case of the Swadesh-based samples where they did converge were very close to

models suffered from various convergence issues, so they are not a viable option to use as the main models, and we do not compare them directly to the main models here in terms of performance (e.g., based on AIC/BIC). Nevertheless, these models had very similar results as the main models, which provides support for the key findings.

Specifically, Table 13 contains these models for the Swadesh-based samples. Both models had results that are extremely similar to the main models, particularly in the case of the key variables that the study focuses on (*distance* and the *distance:proficiency* interaction). The sample for the first subcorpus converged with a "NA/NaN function evaluation warning".[37]

those of the generalized-Poisson models, and functionally equivalent when it comes to the key variables under consideration (i.e., an IRR of 0.99–1 and an SE ≤.01 for *distance* and the *distance:proficiency* interaction).

[37] See the glmmTMB documentation for a description and discussion of all the convergence warnings and errors mentioned here: http://web.archive.org/web/20210516105444/https://cran.r-project.org/web/packages/glmmTMB/vignettes/troubleshooting.html

Table 13. Results of the generalized Poisson models, for the Swadesh-based samples. The response variable was the rate of use of the target L2 English words (i.e., their count offset by the total number of words in each text). Under *fixed effects*, *distance* is the phonological LDN between each L2 word and its most lexically similar L1 counterpart (originally 0–1, scaled to 0–10), *proficiency* is the EFCAMDAT L2 proficiency level at which the text was written (1–12, corresponding to CEFR A1–B2), and *frequency* is the baseline Zipf frequency of the target word in English (~1–7.5). Under random effects, τ_{00} and τ_{11} respectively represent the SD of the associated random intercepts and slopes, and ρ_{01} represents the correlation between random intercepts and associated random slopes (here, *distance* for *L1*).

	First subcorpus						Second subcorpus					
Predictor	*B*	SE_B	*IRR*	SE_{IRR}	*z*	*p*	*B*	SE_B	*IRR*	SE_{IRR}	*z*	*p*
(Intercept)	-10.28	0.12	0.00	<0.01	-86.73	<.001	-9.73	0.13	0.00	<0.01	-77.08	<.001
Distance	-0.01	0.01	0.99	0.01	-1.39	.165	-0.01	0.01	0.99	0.01	-0.59	.552
Proficiency	-0.05	0.02	0.95	0.02	-2.40	.016	-0.01	0.02	0.99	0.02	-0.67	.504
Frequency	3.29	0.14	26.78	3.78	23.32	<.001	3.09	0.16	21.95	3.52	19.29	<.001
Dist:Prof	0.00	<0.01	1.00	<0.01	0.54	.587	0.00	<0.01	1.00	<0.01	-1.04	.296
Random effects												
Learner_τ_{00}	0.00						0.17					
Task_τ_{00}	0.37						0.30					
Word_τ_{00}	0.37						0.47					
Task:Word_τ_{00}	1.81						1.29					
L1_τ_{00}	0.01						0.03					
L1.Distance_τ_{11}	0.01						0.02					
L1_ρ_{01}	0.76						-0.04					

Table 14 contains the generalized Poisson models for the parallel-based samples. There were more convergence issues here, as the first subcorpus did not converge at all (It had a "gradient function must return a numeric vector of length 13" error, as well as a "NA/NaN function evaluation" warning), and the second subcorpus converged with two warnings ("singular convergence" and a "non-positive-definite Hessian matrix").[38] Nevertheless, the findings of the model that did converge, albeit with warnings, are very similar to those of the associated main model.

[38] As noted previously, see the glmmTMB documentation for a description and discussion of all the convergence warnings and errors mentioned here: http://web.archive.org/web/20210516105444/https://cran.r-project.org/web/packages/glmmTMB/vignettes/troubleshooting.html

Table 14. Results of the generalized Poisson models, for the parallel-based samples. The response variable was the rate of use of the target L2 English words (i.e., their count offset by the total number of words in each text). Under *fixed effects*, *distance* is the phonological LDN between each L2 word and its most lexically similar L1 counterpart (originally 0–1, scaled to 0–10), *proficiency* is the EFCAMDAT L2 proficiency level at which the text was written (1–12, corresponding to CEFR A1–B2), and *frequency* is the baseline Zipf frequency of the target word in English (~1–7.5). Under random effects, τ_{00} and τ_{11} respectively represent the SD of the associated random intercepts and slopes, and ρ_{01} represents the correlation between random intercepts and associated random slopes (here, *distance* for *L1*).

	First subcorpus [a]						Second subcorpus					
Predictor	*B*	SE_B	*IRR*	SE_{IRR}	*z*	*p*	*B*	SE_B	*IRR*	SE_{IRR}	*z*	*p*
(Intercept)							-12.49	0.04	0.00	<0.01	-289.28	<.001
Distance							0.01	0.01	1.01	0.01	1.33	.184
Proficiency							0.04	0.01	1.04	0.01	7.09	<.001
Frequency							2.95	0.04	19.07	0.84	67.23	<.001
Dist:Prof							0.00	<0.01	1.00	<0.01	1.11	.265
Random effects												
Learner_τ_{00}							0.00					
Task_τ_{00}							0.11					
Word_τ_{00}							0.67					
Task:Word_τ_{00}							1.43					
L1_τ_{00}							0.00					
L1.Distance_τ_{11}							0.01					
L1_ρ_{01}							1.00					

[a] There are no results for the model in the first subcorpus since it did not converge, but the table is kept in the same format as for the other models to facilitate comparisons.

In summary, we attempted to build models that use variants of the Poisson distribution that can handle both underdispersion and overdispersion (namely, generalized Poisson models). The resulting models had a number of convergence issues, errors, and warnings, which supports the use of the regular Poisson models as the main models in the study. Nevertheless, the findings in the models that did converge, including those that converged with no warnings (i.e., the Swadesh-based models in the first subcorpus) mirror the findings of the main models, especially with regard to the key variables in the study (the *distance* predictor and the *distance:proficiency* interaction). This was expected, since the main issue with underdispersion are overestimated SEs (Brooks et al., 2017, 2019; Dean & Lundy, 2016; Forthmann & Doebler, 2021; Harris et al., 2012; Hartig, 2021b; Sellers & Morris, 2017), and this is not a problem here, given the very small SEs that were found across all samples. As such, these models provide support for the findings of the main models, and suggest that any potential underdispersion in the data does not substantially change our key findings.

4.5.2 Collinearity

In addition to residual plots, we checked for potential collinearity using the *performance* package in R (Lüdecke et al., 2021).[39] The results of this appear in Figure 7, which contains the *variance inflation factor* (VIF) for the predictors in each model. In all cases, the VIF was minimal (i.e., equal to or very close to 1), which indicates the collinearity was not an issue for the present analyses, especially given the large sample sizes (Morrissey & Ruxton, 2018; O'Brien, 2007; Winter, 2019).

[39] The VIF values were calculated using the *performance* package, and the results were plotted using the base R *barplot* function.

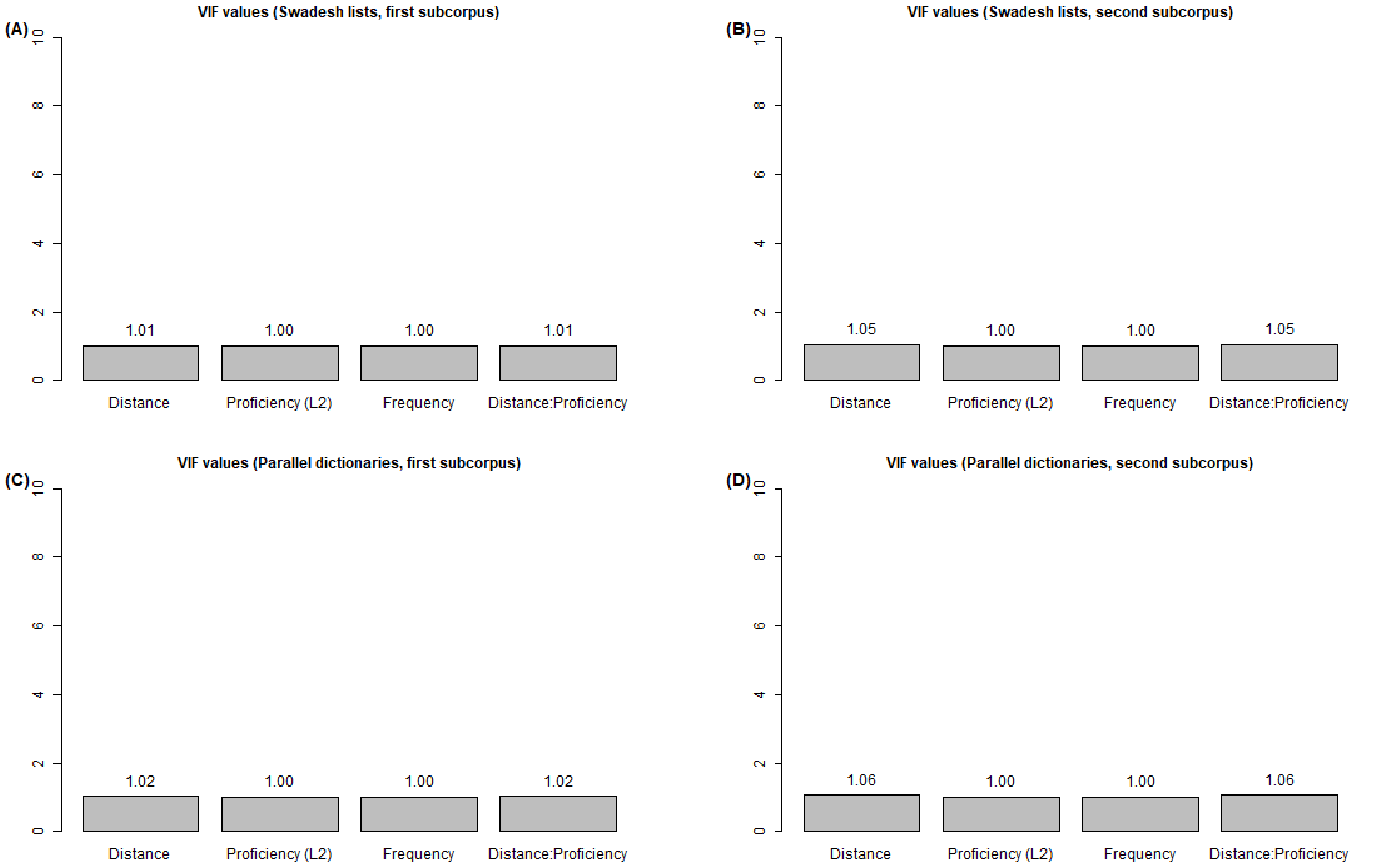

Figure 7. Plots showing the *variance inflation factor* (VIF) for the predictors in each sample, to check for collinearity.

4.6 Software used in the analyses

All analyses were performed in R (R Core Team, 2021).[40] All tests of statistical significance throughout the study were two-tailed. To list the specific packages that were loaded throughout the analyses, we used the *sessionInfo* function from the *report* library (Makowski & Lüdecke, 2019). This generates an automated output based on the citation information associated with the metadata of each package, which may be incomplete or formatted differently than APA style. We kept it here as is, to preserve the original output, and also separated the associated references listed here from the other references used in this document, which appear at the final section of this document.

---*Start of report(sessionInfo()) output below*---

Analyses were conducted using the R Statistical language (version 4.0.4; R Core Team, 2021) on Windows 10 x64 (build 19042), using the packages broom.mixed (version 0.2.6; Ben Bolker and David Robinson, 2020), DHARMa (version 0.4.1; Florian Hartig, 2021), ggplot2 (version 3.3.3; Wickham. ggplot2: Elegant Graphics for Data Analysis. Springer-Verlag New York, 2016.), stringr (version 1.4.0; Hadley Wickham, 2019), forcats (version 0.5.1; Hadley Wickham, 2021), tidyr (version 1.1.3; Hadley Wickham, 2021), readxl (version 1.3.1; Hadley Wickham and Jennifer Bryan, 2019), readr (version 1.4.0; Hadley Wickham and Jim Hester, 2020), dplyr (version 1.0.5; Hadley Wickham et al., 2021), tibble (version 3.1.0; Kirill Müller and Hadley Wickham, 2021), purrr (version 0.3.4; Lionel Henry and Hadley Wickham, 2020), sjPlot (version 2.8.7; Lüdecke D, 2021), performance (version 0.7.0; Lüdecke et al., 2020), glmmTMB (version 1.0.2.1; Mollie Brooks et al., 2017), openxlsx (version 4.2.3; Philipp Schauberger and Alexander Walker, 2020) and tidyverse (version 1.3.0; Wickham et al., 2019).

References

----

[40] However, the lexical-distance data was generated in Python. Specifically, the following Python libraries were used for basic data wrangling and calculations: *SciPy* (Virtanen et al., 2019), *pandas* (McKinney, 2010), and *numpy* (Oliphant, 2006; Walt et al., 2011). The ASJP's phonetic script (outlined in Brown et al., 2008) was converted to IPA using the *asjp* library (Sofroniev, 2018). Distances were calculated using the *PanPhon* library (Mortensen et al., 2016).

- Ben Bolker and David Robinson (2020). broom.mixed: Tidying Methods for Mixed Models. R package version 0.2.6. https://CRAN.R-project.org/package=broom.mixed

- Florian Hartig (2021). DHARMa: Residual Diagnostics for Hierarchical (Multi-Level / Mixed) Regression Models. R package version 0.4.1. https://CRAN.R-project.org/package=DHARMa

- H. Wickham. ggplot2: Elegant Graphics for Data Analysis. Springer-Verlag New York, 2016.

- Hadley Wickham (2019). stringr: Simple, Consistent Wrappers for Common String Operations. R package version 1.4.0. https://CRAN.R-project.org/package=stringr

- Hadley Wickham (2021). forcats: Tools for Working with Categorical Variables (Factors). R package version 0.5.1. https://CRAN.R-project.org/package=forcats

- Hadley Wickham (2021). tidyr: Tidy Messy Data. R package version 1.1.3. https://CRAN.R-project.org/package=tidyr

- Hadley Wickham and Jennifer Bryan (2019). readxl: Read Excel Files. R package version 1.3.1. https://CRAN.R-project.org/package=readxl

- Hadley Wickham and Jim Hester (2020). readr: Read Rectangular Text Data. R package version 1.4.0. https://CRAN.R-project.org/package=readr

- Hadley Wickham, Romain François, Lionel Henry and Kirill Müller (2021). dplyr: A Grammar of Data Manipulation. R package version 1.0.5. https://CRAN.R-project.org/package=dplyr

- Kirill Müller and Hadley Wickham (2021). tibble: Simple Data Frames. R package version 3.1.0. https://CRAN.R-project.org/package=tibble

- Lionel Henry and Hadley Wickham (2020). purrr: Functional Programming Tools. R package version 0.3.4. https://CRAN.R-project.org/package=purrr

- Lüdecke D (2021). _sjPlot: Data Visualization for Statisticsin Social Science_. R package version 2.8.7, <URL:https://CRAN.R-project.org/package=sjPlot>.

- Lüdecke, Makowski, Waggoner & Patil (2020). Assessment of Regression Models Performance. CRAN. Available from https://easystats.github.io/performance/

- Mollie E. Brooks, Kasper Kristensen, Koen J. van Benthem, Arni Magnusson, Casper W. Berg, Anders Nielsen, Hans J. Skaug, Martin Maechler and Benjamin M. Bolker (2017).

glmmTMB Balances Speed and Flexibility Among Packages for Zero-inflated Generalized Linear Mixed Modeling. The R Journal, 9(2), 378-400.

- Philipp Schauberger and Alexander Walker (2020). openxlsx: Read, Write and Edit xlsx Files. R package version 4.2.3. https://CRAN.R-project.org/package=openxlsx

- R Core Team (2021). R: A language and environment for statistical computing. R Foundation for Statistical Computing, Vienna, Austria. URL https://www.R-project.org/.

- Wickham et al., (2019). Welcome to the tidyverse. Journal of Open Source Software, 4(43), 1686, https://doi.org/10.21105/joss.01686

---*End of report(sessionInfo()) output above*---

5 APPENDIX S5: ADDITIONAL MODELS

5.1 Models with alternative random effects

5.1.1 Random slopes

Initially, we tested several potential mixed-effects models, with random slopes of *lexical distance* for the *learner*, *L1*, *task*, and *word* random effects (separately for each one). For the models based on the parallel dictionaries, only the model with random slopes for *L1* converged properly, as the other models either had problems with singular convergence or did not converge at all, even though they were tested on their own (i.e., as a single random slope, before combining multiple ones).

Given this, and given that the goal was to use a consistent random-effects structure across all models, we included only random slopes of *distance* for *L1* in these models. However, as shown in the results section of the main paper, this does not appear to be an issue given our particular findings, since the main concern with omitting random slopes is an increased rate of Type I error (Matuschek et al., 2017; Winter, 2019), but our key findings provide support for the null hypothesis.

5.1.2 Random intercepts by text

We considered adding to the models a random effect (random intercepts) for each *text* in the sample. However, there is substantial overlap between this and the *learner* random effect, since, as noted in the paper, most learners only had a single text in the sample.[41] In addition, we also had the *task* random effect, which accounts for further variance that may be associated with specific texts (each learner had only a single text per task).

When we attempted to build models that included the *text* random effect in addition to *learner*, in the case of the parallel-based models, the model did not converge for the first subcorpus, and had convergence warning for the second subcorpus.[42] Given this, and given that the goal was to use a consistent random-effects structure across all models, we did not include this random effect in our final models.

[41] The mean number of texts per learner was 1.36 in the first subcorpus and 1.41 in the second. For more details on this, see the "Sample information" document in the study's OSF repository.
[42] In the first subcorpus, we had a "gradient function must return a numeric vector of length 8" error, as well as "NA/NaN function evaluation" and "restarting interrupted promise evaluation" warnings. In the second subcorpus, we had the same warnings as in the first subcorpus, but not the error.

Nevertheless, as shown Tables 15 and 16, the models that did converge with this random effect were functionally equivalent to the models without it, so excluding this effect from the main models does not make a substantial difference to our findings.[43]

[43] In addition, note that the random effect of *text* was estimated to be functionally equivalent to zero in 2 out of the 3 models that did converge, possibly because there was not sufficient information to disentangle it from the *learner* random effect.

Table 15. Results of the mixed-models with *text* as an additional random effect, for the Swadesh-based samples. The response variable was the rate of use of the target L2 English words (i.e., their count offset by the total number of words in each text). Under *fixed effects*, *distance* is the phonological LDN between each L2 word and its most lexically similar L1 counterpart (originally 0–1, scaled to 0–10), *proficiency* is the EFCAMDAT L2 proficiency level at which the text was written (1–12, corresponding to CEFR A1–B2), and *frequency* is the baseline Zipf frequency of the target word in English (~1–7.5). Under random effects, τ_{00} and τ_{11} respectively represent the SD of the associated random intercepts and slopes, and ρ_{01} represents the correlation between random intercepts and associated random slopes (here, *distance* for *L1*).

	First subcorpus						Second subcorpus					
Predictor	*B*	SE_B	*IRR*	SE_{IRR}	*z*	*p*	*B*	SE_B	*IRR*	SE_{IRR}	*z*	*p*
(Intercept)	-10.32	0.16	0.00	<0.01	-65.39	<.001	-9.87	0.14	0.00	<0.01	-68.53	<.001
Distance	-0.01	0.01	0.99	0.01	-1.17	.243	-0.01	0.01	0.99	0.01	-0.38	.701
Proficiency	-0.04	0.02	0.96	0.02	-2.12	.034	0.00	0.02	1.00	0.02	-0.25	.802
Frequency	3.30	0.21	26.99	5.66	15.70	<.001	3.16	0.19	23.50	4.50	16.49	<.001
Dist:Prof	0.00	<0.01	1.00	<0.01	0.61	.543	0.00	<0.01	1.00	<0.01	-1.18	.238
Random effects												
Learner_τ_{00}	0.07						0.15					
Text_τ_{00}	0.00						0.24					
Task_τ_{00}	0.40						0.33					
Word_τ_{00}	0.38						0.46					
Task:Word_τ_{00}	1.84						1.36					
L1_τ_{00}	0.02						0.03					
L1.Distance_τ_{11}	0.01						0.03					
L1_ρ_{01}	0.55						-0.05					

Table 16. Results of the mixed-models with *text* as an additional random effect, for the parallel-based samples. The response variable was the rate of use of the target L2 English words (i.e., their count offset by the total number of words in each text). Under *fixed effects*, *distance* is the phonological LDN between each L2 word and its most lexically similar L1 counterpart (originally 0–1, scaled to 0–10), *proficiency* is the EFCAMDAT L2 proficiency level at which the text was written (1–12, corresponding to CEFR A1–B2), and *frequency* is the baseline Zipf frequency of the target word in English (~1–7.5). Under random effects, τ_{00} and τ_{11} respectively represent the SD of the associated random intercepts and slopes, and ρ_{01} represents the correlation between random intercepts and associated random slopes (here, *distance* for *L1*).

	First subcorpus						Second subcorpus					
Predictor	*B*	SE_B	*IRR*	SE_{IRR}	*z*	*p*	*B*	SE_B	*IRR*	SE_{IRR}	*z*	*p*
(Intercept)							-12.59	0.05	0.00	<0.01	-243.41	<.001
Distance							0.01	0.01	1.01	0.01	1.04	.301
Proficiency							0.04	0.01	1.04	0.01	4.29	<.001
Frequency							2.97	0.05	19.50	0.99	58.52	<.001
Dist:Prof							0.00	<0.01	1.00	<0.01	1.09	.276
Random effects												
Learner_τ_{00}							0.04					
Text_τ_{00}							0.00					
Task_τ_{00}							0.11					
Word_τ_{00}							0.65					
Task:Word_τ_{00}							1.50					
L1_τ_{00}							0.01					
L1.Distance_τ_{11}							0.01					
L1_ρ_{01}							0.81					

5.1.3 Models without the L1 random effect

We built supplementary models without the L1 random effect (i.e., without random slopes of *distance* for *L1* and without random intercepts for *L1*). The main reason for this is that there was only a relatively small number of L1s in the samples, particularly in the parallel-based models, so we wanted to check whether and how removing this effect would change the estimates for the other effects.

As shown Tables 17 and 18, the findings of these models largely replicate those of our main models, which indicates that including or excluding the L1 random effect does not substantially influence the findings.

Table 17. Results of the mixed-models without the *L1* random effect, for the Swadesh-based samples. The response variable was the rate of use of the target L2 English words (i.e., their count offset by the total number of words in each text). Under *fixed effects*, *distance* is the phonological LDN between each L2 word and its most lexically similar L1 counterpart (originally 0–1, scaled to 0–10), *proficiency* is the EFCAMDAT L2 proficiency level at which the text was written (1–12, corresponding to CEFR A1–B2), and *frequency* is the baseline Zipf frequency of the target word in English (~1–7.5). Under random effects, τ_{00} represents the SD of the associated random intercepts.

	First subcorpus						Second subcorpus					
Predictor	*B*	SE_B	*IRR*	SE_{IRR}	*z*	*p*	*B*	SE_B	*IRR*	SE_{IRR}	*z*	*p*
(Intercept)	-10.32	0.16	0.00	<0.01	-65.41	<.001	-9.85	0.14	0.00	<0.01	-68.76	<.001
Distance	-0.01	0.01	0.99	0.01	-2.05	.040	-0.01	0.01	0.99	0.01	-0.99	.321
Proficiency	-0.04	0.02	0.96	0.02	-2.12	.034	0.00	0.02	1.00	0.02	-0.22	.827
Frequency	3.29	0.21	26.97	5.66	15.70	<.001	3.15	0.19	23.40	4.46	16.52	<.001
Dist:Prof	0.00	<0.01	1.00	<0.01	0.72	.471	0.00	<0.01	1.00	<0.01	-1.25	.211
Random effects												
Learner_τ_{00}	0.07						0.24					
Task_τ_{00}	0.40						0.33					
Word_τ_{00}	0.38						0.46					
Task:Word_τ_{00}	1.84						1.36					

Table 18. Results of the mixed-models without the *L1* random effect, for the parallel-based samples. The response variable was the rate of use of the target L2 English words (i.e., their count offset by the total number of words in each text). Under *fixed effects*, *distance* is the phonological LDN between each L2 word and its most lexically similar L1 counterpart (originally 0–1, scaled to 0–10), *proficiency* is the EFCAMDAT L2 proficiency level at which the text was written (1–12, corresponding to CEFR A1–B2), and *frequency* is the baseline Zipf frequency of the target word in English (~1–7.5). Under random effects, τ_{00} represents the SD of the associated random intercepts.

	First subcorpus						Second subcorpus					
Predictor	*B*	SE_B	*IRR*	SE_{IRR}	*z*	*p*	*B*	SE_B	*IRR*	SE_{IRR}	*z*	*p*
(Intercept)	-12.84	0.06	0.00	<0.01	-210.05	<.001	-12.58	0.05	0.00	<0.01	-246.85	<.001
Distance	0.01	<0.01	1.01	<0.01	3.44	.001	0.01	<0.01	1.01	<0.01	1.54	.124
Proficiency	0.12	0.01	1.13	0.01	10.17	<.001	0.04	0.01	1.04	0.01	4.21	<.001
Frequency	2.90	0.06	18.15	1.05	49.97	<.001	2.97	0.05	19.48	0.99	58.51	<.001
Dist:Prof	0.00	<0.01	1.00	<0.01	1.21	.226	0.00	<0.01	1.00	<0.01	1.07	.283
Random effects												
Learner_τ_{00}	0.03						0.05					
Task_τ_{00}	0.03						0.11					
Word_τ_{00}	0.44						0.65					
Task:Word_τ_{00}	2.32						1.50					

5.2 Baseline models (without distance)

The baseline models were models that did not include lexical distance at all (MODELS$_{baseline}$). We compared these models to the main models that were used in the study (MODELS$_{main}$), where lexical distance was included as a predictor, as part of an interaction with L2 proficiency, and as random slopes of L1. In addition, to better understand how the removal of lexical distance from the models influences them,[44] we also compared the baseline and main models with models that contained distance as a predictor/interaction but without random slopes (MODELS$_{no_slope}$), and with models that had distance only as a predictor, with no random slopes or interaction (MODELS$_{only_predictor}$).

Specifically, we compared the models' AIC and BIC, and the results of this are shown in Table 19. Both measures were used, as suggested in Kuha, (2004). The AIC and BIC of each model were extracted directly from each model object in R using the *summary* function. All comparisons were between models that used the same set of data (i.e., between models that use the same learner sample and lexical-distance dataset), as required when using these measures (Fabozzi et al., 2014; Kuha, 2004).

[44] For example, this could show if removing lexical distance from the interaction improves the models, but removing it as a predictor worsens them.

Table 19. Comparisons of AIC and BIC across models.

Subcorpus	Distance data	Model	AIC	Δ AIC	BIC	Δ BIC
First	Swadesh	baseline	73703.03	0.58	73785.16	-
First	Swadesh	only predictor	73702.45	-	73794.85	9.69
First	Swadesh	no slope	73704.03	1.59	73806.70	21.54
First	Swadesh	main	73705.32	2.87	73828.52	43.36
Second	Swadesh	baseline	61475.10	5.66	61554.95	-
Second	Swadesh	only predictor	61474.56	5.11	61564.39	9.44
Second	Swadesh	no slope	61474.99	5.55	61574.80	19.86
Second	Swadesh	main	61469.44	-	61589.22	34.27
First	parallel	baseline	401614.39	-	401722.16	-
First	parallel	only predictor	401663.18	48.80	401784.42	62.27
First	parallel	no slope	401662.38	48.00	401797.09	74.94
First	parallel	main	401655.51	41.12	401817.16	95.01
Second	parallel	baseline	346322.57	5.27	346428.02	-
Second	parallel	only predictor	346322.72	5.41	346441.34	13.33
Second	parallel	no slope	346323.49	6.18	346455.29	27.28
Second	parallel	main	346317.31	-	346475.47	47.46

Note. ΔAIC is calculated by subtracting the AIC of a given model from the AIC of the model with the minimal AIC for that combination of subcorpus (i.e., first/second) and lexical-distance dataset (i.e., Swadesh/parallel), since comparisons can only be made between models that are based on the same data (Fabozzi et al., 2014; Kuha, 2004). Accordingly, no ΔAIC is listed for the model with the minimal AIC for a certain combination (e.g., Swadesh lists in the first subcorpus). The same is the case for ΔBIC.

Interpretations of the differences in AIC/BIC are based on Fabozzi et al. (2014). In terms of BIC, there was very strong support for the simplest (baseline) model in all 4 cases, as it had the minimal BIC, with ΔBIC either slightly below 10 or far above it. In terms of AIC, the picture was less clear. Specifically, in the case of the parallel dictionaries in the first subcorpus, the baseline model was strongly supported (ΔAIC > 40). However, in the case the first subcorpus in the Swadesh lists, there was only weak support for the baseline and predictor-only models over the main model (ΔAIC ~2–3), and in the case of the second

subcorpus (both Swadesh and parallel), there was moderate support (ΔAIC ~5–6) for the main models over the other models (though the main models were ranked the worst in all cases based on BIC). This difference between AIC/BIC can be attributed to the greater penalty that BIC imposes for the number of parameters in the model (Fabozzi et al., 2014). When the patterns of the two measures are considered, together with the estimates for the associated predictors, it appears that the AIC comparisons are sometimes recommending the use of an overfitted model here.

Overall, the comparison between the models did not consistently support the inclusion of linguistic distance as a predictor based on AIC, and consistently supported its exclusion based on BIC. It is, therefore, reasonable to conclude that the effect of distance is at best unclear in our dataset. This is strongly supported by the findings for the main models that are shown in the paper, where the distance predictor and the interaction had IRRs very close to 1 (corresponding to a coefficient estimate of 0) and very small SEs, and where the SDs of the random slopes of distance were also very close to 1 (i.e., to a coefficient estimate of 0).

The results for the baseline models are shown in Tables 20 and 21.

Table 20. Results of the baseline mixed-effects models, for the Swadesh-based samples. The response variable was the rate of use of the target L2 English words (i.e., their count offset by the total number of words in each text). Under *fixed effects*, *proficiency* is the EFCAMDAT L2 proficiency level at which the text was written (1–12, corresponding to CEFR A1–B2), and *frequency* is the baseline Zipf frequency of the target word in English (~1–7.5). Under random effects, τ_{00} represents the standard deviation (SD) of the associated random intercepts.

	First subcorpus						Second subcorpus					
Predictor	*B*	SE_B	*IRR*	SE_{IRR}	*z*	*p*	*B*	SE_B	*IRR*	SE_{IRR}	*z*	*p*
(Intercept)	-10.32	0.16	0.00	<0.01	-65.30	<.001	-9.85	0.14	0.00	<0.01	-68.62	<.001
Proficiency	-0.04	0.02	0.96	0.02	-2.11	.035	0.00	0.02	1.00	0.02	-0.25	.806
Frequency	3.29	0.21	26.89	5.65	15.68	<.001	3.15	0.19	23.31	4.44	16.52	<.001
Random effects												
Learner_τ_{00}	0.07						0.23					
Task_τ_{00}	0.40						0.33					
Word_τ_{00}	0.38						0.46					
Task:Word_τ_{00}	1.84						1.36					
L1_τ_{00}	0.02						0.03					

Table 21. Results of the baseline mixed-effects models, for the parallel-based samples. The response variable was the rate of use of the target L2 English words (i.e., their count offset by the total number of words in each text). Under *fixed effects*, *proficiency* is the EFCAMDAT L2 proficiency level at which the text was written (1–12, corresponding to CEFR A1–B2), and *frequency* is the baseline Zipf frequency of the target word in English (~1–7.5). Under random effects, τ_{00} represents the standard deviation (SD) of the associated random intercepts.

	First subcorpus						Second subcorpus					
Predictor	*B*	SE_B	*IRR*	SE_{IRR}	*z*	*p*	*B*	SE_B	*IRR*	SE_{IRR}	*z*	*p*
(Intercept)	-12.86	0.06	0.00	<0.01	-207.24	<.001	-12.59	0.05	0.00	<0.01	-242.46	<.001
Proficiency	0.11	0.01	1.11	0.01	9.02	<.001	0.04	0.01	1.04	0.01	4.36	<.001
Frequency	2.90	0.06	18.14	1.05	49.90	<.001	2.97	0.05	19.57	0.99	58.57	<.001
Random effects												
Learner_τ_{00}	0.03						0.05					
Task_τ_{00}	0.03						0.12					
Word_τ_{00}	0.46						0.65					
Task:Word_τ_{00}	2.30						1.50					
L1_τ_{00}	0.01						0.01					

5.3 German-only models

We built models using data from only German learners.[45] The focus on German was because it was the closest L1 to English, and it had the broadest distribution of LDN scores, especially in the Swadesh lists. This style of analysis is also similar to the analyses used by some other researchers, assessed and found L2 cognate facilitation among speakers of a single L1 that is similar to the target L2 (e.g., De Wilde et al., 2020, who looked at L2 English among L1 Dutch speakers).

There were 950 texts written by German speakers in the first subcorpus and 710 such texts in the second subcorpus. The results of the associated models, which are shown in Tables 22 and 23, replicate the key findings of the main models. Specifically, the effect of distance is insignificant and inconsistent across all the models (B = -0.03–0.04 and IRR = 0.97–1.04, p = .123–.885). The interaction between distance and L2 proficiency is significant here for some (3) of the models, unlike in the main models, despite the smaller sample size, since it is a tiny bit stronger here, but the effect size is so weak that it is still functionally identical to a null effect (B = 0.01–0.02 and IRR = 1.01–1.02, compared to B = 0.00 and IRR = 1.00). In addition, the random effects of *task*, *word*, and *task:word* were strong here, similarly to the main models.[46]

In summary, the samples using only data from German speakers largely replicated our key findings, which indicates that our findings hold even when focusing on this key L1.

[45] There is no L1 random effect in these models, since there is only a single L1 in the models. Nevertheless, as shown in the "Models without the L1 random effect" section of this appendix, removing this effect does not change the results of the main models, given how weak it is.

[46] The one notable difference is the much weaker effect of frequency in the parallel-based model in the first corpus, together with an associated increase in the magnitude of the intercept. We do not have a clear explanation for this, but it is not crucial for the present analyses, given that the key findings replicate despite of this, and that this was an issue for only one of the four models.

Table 22. Results of the mixed-models without the *L1* random effect, for the Swadesh-based samples, using only data from German speakers. The response variable was the rate of use of the target L2 English words (i.e., their count offset by the total number of words in each text). Under *fixed effects*, *distance* is the phonological LDN between each L2 word and its most lexically similar L1 counterpart (originally 0–1, scaled to 0–10), *proficiency* is the EFCAMDAT L2 proficiency level at which the text was written (1–12, corresponding to CEFR A1–B2), and *frequency* is the baseline Zipf frequency of the target word in English (~1–7.5). Under random effects, τ_{00} represents the SD of the associated random intercepts.

	First subcorpus						Second subcorpus					
Predictor	*B*	SE_B	*IRR*	SE_{IRR}	*z*	*p*	*B*	SE_B	*IRR*	SE_{IRR}	*z*	*p*
(Intercept)	-9.81	0.23	0.00	<0.01	-43.38	<.001	-9.33	0.19	0.00	<0.01	-50.06	<.001
Distance	0.04	0.07	1.04	0.07	0.51	.607	0.01	0.06	1.01	0.06	0.22	.828
Proficiency	-0.07	0.02	0.93	0.02	-3.20	.001	-0.01	0.02	0.99	0.02	-0.34	.732
Frequency	2.66	0.25	14.35	3.62	10.56	<.001	2.67	0.20	14.43	2.89	13.34	<.001
Dist:Prof	0.02	0.01	1.02	0.01	2.41	.016	0.01	0.01	1.01	0.01	1.34	.180
Random effects												
Learner_τ_{00}	0.00						0.27					
Task_τ_{00}	0.13						0.20					
Word_τ_{00}	0.21						0.30					
Task:Word_τ_{00}	1.79						1.15					

Table 23. Results of the mixed-models without the *L1* random effect, for the parallel-based samples, using only data from German speakers. The response variable was the rate of use of the target L2 English words (i.e., their count offset by the total number of words in each text). Under *fixed effects*, *distance* is the phonological LDN between each L2 word and its most lexically similar L1 counterpart (originally 0–1, scaled to 0–10), *proficiency* is the EFCAMDAT L2 proficiency level at which the text was written (1–12, corresponding to CEFR A1–B2), and *frequency* is the baseline Zipf frequency of the target word in English (~1–7.5). Under random effects, τ_{00} represents the SD of the associated random intercepts.

	First subcorpus						Second subcorpus					
Predictor	*B*	SE_B	*IRR*	SE_{IRR}	*z*	*p*	*B*	SE_B	*IRR*	SE_{IRR}	*z*	*p*
(Intercept)	-15.46	0.07	0.00	<0.01	-235.98	<.001	-12.47	0.06	0.00	<0.01	-205.52	<.001
Distance	0.00	0.02	1.00	0.02	-0.14	.885	-0.03	0.02	0.97	0.02	-1.54	.123
Proficiency	-0.03	0.01	0.97	0.01	-3.06	.002	0.02	0.01	1.02	0.01	2.62	.009
Frequency	0.13	0.06	1.14	0.07	2.01	.044	2.65	0.06	14.22	0.85	44.48	<.001
Dist:Prof	0.02	<0.01	1.02	<0.01	6.69	<.001	0.01	<0.01	1.01	<0.01	2.64	.008
Random effects												
Learner_τ_{00}	0.00						0.03					
Task_τ_{00}	0.03						0.03					
Word_τ_{00}	0.34						0.38					
Task:Word_τ_{00}	2.29						1.66					

5.4 Binary-response models

Since the underlying response variable that we examined is a *count*, we used Poisson models for our main analyses (Green, 2021; Hox et al., 2018; Winter, 2019). Nevertheless, since lexical distance might affect whether or not an L2 word is used at all, rather than how often it is used, we also built supplementary logistic-regression models with a binary response variable.

We derived the binary response variable by converting the original count of the number of times that each target English word was used in a text to a binary variable (i.e., a count greater than 0 was converted to a '1' in the response variable, and a count of 0 was kept as a zero). To model this response variable, we used logistic regression (i.e., models with the *binomial* family and canonical *logit* link). The total wordcount of texts was included in the models as a direct predictor, similarly to the offset in the Poisson models. We also included exponentiated coefficients, which in this case are called *odds ratio* (OR) rather than *incidence rate ratio* in Poisson models, though the two are similar conceptually (e.g., an OR of 2 means that a 1-unit increase in the predictor doubles the likelihood that the target word will be used in a text).

There was an issue with singular convergence in the Swadesh-based sample in the first subcorpus, due to the L1 random effect, and the parallel-based models did not converge at all,[47] so we omitted this random effect from the models. However, given the correspondence between these models and the Poisson models (as shown below), and given that removing this random effect from the Poisson models did not change the key findings (see "Models without the L1 random effect in the Supporting Information"), this should not substantially influence our key findings. Indeed, for the Swadesh models that did converge (albeit with singular convergence), the results of the models were functionally identical to the Swadesh models without the L1 random effect.

The results of binary-response (i.e., logistic-regression) models appear in Tables 24 and 25. Despite reducing the complexity of the models by removing the L1 random effect, the model still did not converge for the parallel-based sample in the first corpus, which supports our preference for using the Poisson models as the main models. Nevertheless, the three models that did converge without an issue replicate the results of our main models, as

[47] Specifically, they had a "gradient function must return a numeric vector of length 7" error and a "NA/NaN function evaluation" warning.

there was a functionally null effect of distance and of its interaction with proficiency (*B* = -0.02–0.00, corresponding to *OR* = 0.98–1.00) as well as strong effects of *task*, *word*, and especially the *task:word* interaction. This means that looking at a binary response variable (whether a word was or was not used in a text), rather than a count response variable (the number of time a word was used in a text), does not change our findings substantially.

Table 24. Results of the mixed-models with a binary response variable (i.e., whether a target English word did or did not appear in the text), for the Swadesh-based samples. Under *fixed effects*, *distance* is the phonological LDN between each L2 word and its most lexically similar L1 counterpart (originally 0–1, scaled to 0–10), *proficiency* is the EFCAMDAT L2 proficiency level at which the text was written (1–12, corresponding to CEFR A1–B2), and *frequency* is the baseline Zipf frequency of the target word in English (~1–7.5). Under random effects, τ_{00} represents the SD of the associated random intercepts.

	First subcorpus						Second subcorpus					
Predictor	*B*	SE_B	*OR*	SE_{OR}	*z*	*p*	*B*	SE_B	*OR*	SE_{OR}	*z*	*p*
(Intercept)	-7.43	0.20	0.00	<0.01	-38.08	<.001	-6.62	0.18	0.00	<0.01	-37.63	<.001
Distance	-0.02	0.01	0.98	0.01	-1.99	.046	-0.01	0.01	0.99	0.01	-0.95	.344
Proficiency	-0.04	0.03	0.96	0.02	-1.78	.075	0.00	0.02	1.00	0.02	-0.03	.979
Frequency	3.74	0.25	42.21	10.39	15.21	<.001	3.49	0.22	32.63	7.12	15.98	<.001
Wordcount	0.01	<0.01	1.01	<0.01	18.09	<.001	0.01	<0.01	1.01	<0.01	15.84	<.001
Dist:Prof	0.00	<0.01	1.00	<0.01	0.49	.625	0.00	<0.01	1.00	<0.01	-0.55	.585
Random effects												
Learner_τ_{00}	0.16						0.20					
Task_τ_{00}	0.44						0.39					
Word_τ_{00}	0.40						0.53					
Task:Word_τ_{00}	2.25						1.49					

Table 25. Results of the mixed-models with a binary response variable (i.e., whether a target English word did or did not appear in the text), for the Swadesh-based samples. Under fixed effects, distance is the phonological LDN between each L2 word and its most lexically similar L1 counterpart (originally 0–1, scaled to 0–10), proficiency is the EFCAMDAT L2 proficiency level at which the text was written (1–12, corresponding to CEFR A1–B2), and frequency is the baseline Zipf frequency of the target word in English (~1–7.5). Under random effects, τ_{00} represents the SD of the associated random intercepts.

	First subcorpus [a]						Second subcorpus					
Predictor	*B*	SE_B	*OR*	SE_{OR}	*z*	*p*	*B*	SE_B	*OR*	SE_{OR}	*z*	*p*
(Intercept)							-9.16	0.06	0.00	<0.01	-149.88	<.001
Distance							0.00	<0.01	1.00	<0.01	-0.75	.455
Proficiency							0.03	0.01	1.03	0.01	2.57	.010
Frequency							3.05	0.05	21.10	1.09	59.06	<.001
Wordcount							0.01	<0.01	1.01	<0.01	35.09	<.001
Dist:Prof							0.00	<0.01	1.00	<0.01	1.22	.224
Random effects												
Learner_τ_{00}							0.11					
Task_τ_{00}							0.16					
Word_τ_{00}							0.66					
Task:Word_τ_{00}							1.53					

[a] There are no results for the model in the first subcorpus since it did not converge, but the table is kept in the same format as for the other models to facilitate comparisons.

5.5 Added-interactions models

Our main models included an interaction between distance and L2 proficiency, since that was the key interaction that we expected to find based on the literature (specifically, we expected that the effects of distance will weaken as L2 proficiency increases). To expand these models, we created supplementary models with three additional interactions:

- The first interaction was for *distance* and *frequency*, primarily in case the effects of distance are stronger in lower-frequency words (e.g., because those words are more difficult, so learners rely more on the facilitative effects of similarity, or because these words are more likely to be a part of an interchangeable synonym set).
- The second interaction was for *proficiency* and *frequency* (e.g., if proficiency effects are stronger for lower-frequency words, that people are less likely to know).
- The third interaction was an interaction between *distance, proficiency,* and *frequency*. Such three-way interaction is more difficult to interpret, but one way to think of it is that the interaction between distance and proficiency, if it exists, may itself be moderated by the frequency of the words (e.g., the effect of distance is weaker as L2 proficiency increases, but only for high-frequency words).

These interactions were inserted into the models in R by specifying:

```
ldn_phono_closest_scaled_centered *
proficiency_level_centered *
frequency_zipf_centered
```

To accommodate the extra complexity of these models, we removed the L1 random effect, which was the key cause of convergence issues in the other models, and which did not substantially influence the findings (see the “Models without the L1 random effect” section in this Appendix).

The results of these models are shown in Tables 26 and 27. The models converged with no issues, except for the parallel-based sample in the first corpus where the model did not converge.[48]

[48] Specifically, the model that did not converge had a “gradient function must return a numeric vector of length 4” error and a “NA/NaN function evaluation” warning.

The models that converged all replicated the key findings of the main models, in terms of a null effect of the distance predictor, the null interaction between distance and proficiency, and the strong effects of the *task, word*, and *task:word*.

In addition, the added two-way interaction between distance and frequency, and the added three-way interaction between distance, proficiency, and frequency were consistently and robustly null across all the models (B = -.0.02–0.01 and $SE_B \leq 0.01$, corresponding to IRR = 0.98–1.01 and $SE_{IRR} \leq 0.01$). Conversely, there was a consistent, positive, but weak interaction between proficiency and frequency (B = 0.05–0.07, corresponding to IRR = 1.05–1.07), which suggests that as learners' L2 proficiency increases, the effect of word frequency on their rate of use of the L2 words also increases. However, this small interaction is irrelevant to the present research, and as shown in the models, it does not change the key findings.

In summary, we found no substantial interaction between distance and other predictors (i.e., between distance/proficiency, distance/frequency, and distance/proficiency/frequency). In addition, our key findings replicate when adding the new interactions into our models, although adding this interaction does cause convergence issues. This does not suggest that these interactions cannot occur in other contexts; for example, there may indeed be a distance/proficiency and a distance/frequency interaction in more spontaneous L2 settings, where there is a stronger effect of distance. Rather, this merely indicates that these interactions did not occur in the present sample, likely due to the general lack of effect of distance.

Table 26. Results of the mixed-models with the added interactions, for the Swadesh-based samples. The response variable was the rate of use of the target L2 English words (i.e., their count offset by the total number of words in each text). Under *fixed effects*, *distance* is the phonological LDN between each L2 word and its most lexically similar L1 counterpart (originally 0–1, scaled to 0–10), *proficiency* is the EFCAMDAT L2 proficiency level at which the text was written (1–12, corresponding to CEFR A1–B2), and *frequency* is the baseline Zipf frequency of the target word in English (~1–7.5). Under random effects, τ_{00} represents the SD of the associated random intercepts.

	First subcorpus						Second subcorpus					
Predictor	*B*	SE_B	*IRR*	SE_{IRR}	*z*	*p*	*B*	SE_B	*IRR*	SE_{IRR}	*z*	*p*
(Intercept)	-10.31	0.16	0.00	<0.01	-65.58	<.001	-9.82	0.14	0.00	<0.01	-69.16	<.001
Distance	0.00	0.01	1.00	0.01	0.23	.820	-0.01	0.01	0.99	0.01	-0.49	.623
Proficiency	-0.06	0.02	0.94	0.02	-2.80	.005	-0.03	0.02	0.97	0.02	-1.52	.128
Frequency	3.29	0.21	26.77	5.60	15.72	<.001	3.12	0.19	22.59	4.28	16.47	<.001
Dist:Prof	0.00	<0.01	1.00	<0.01	0.77	.444	0.00	<0.01	1.00	<0.01	-0.69	.492
Dist:Freq	-0.02	0.01	0.98	0.01	-2.28	.023	0.00	0.01	1.00	0.01	-0.14	.887
Prof:Freq	0.05	0.02	1.05	0.02	2.15	.032	0.06	0.02	1.06	0.02	3.10	.002
Dist:Prof:Freq	0.00	<0.01	1.00	<0.01	-0.23	.819	0.00	<0.01	1.00	<0.01	-0.30	.764
Random effects												
Learner_τ_{00}	0.07						0.24					
Task_τ_{00}	0.40						0.33					
Word_τ_{00}	0.38						0.45					
Task:Word_τ_{00}	1.83						1.35					

Table 27. Results of the mixed-models with the added interactions, for the parallel-based samples. The response variable was the rate of use of the target L2 English words (i.e., their count offset by the total number of words in each text). Under *fixed effects*, *distance* is the phonological LDN between each L2 word and its most lexically similar L1 counterpart (originally 0–1, scaled to 0–10), *proficiency* is the EFCAMDAT L2 proficiency level at which the text was written (1–12, corresponding to CEFR A1–B2), and *frequency* is the baseline Zipf frequency of the target word in English (~1–7.5). Under random effects, τ_{00} represents the SD of the associated random intercepts.

	First subcorpus [a]						Second subcorpus					
Predictor	*B*	SE_B	*IRR*	SE_{IRR}	*z*	*p*	*B*	SE_B	*IRR*	SE_{IRR}	*z*	*p*
(Intercept)							-12.49	0.05	0.00	<0.01	-247.58	<.001
Distance							-0.01	0.01	0.99	0.01	-1.93	.054
Proficiency							-0.02	0.01	0.98	0.01	-2.19	.029
Frequency							2.89	0.05	17.94	0.91	57.01	<.001
Dist:Prof							0.00	<0.01	1.00	<0.01	-1.27	.206
Dist:Freq							0.01	<0.01	1.01	<0.01	3.05	.002
Prof:Freq							0.07	0.01	1.07	0.01	12.81	<.001
Dist:Prof:Freq							0.00	<0.01	1.00	<0.01	1.72	.086
Random effects												
Learner_τ_{00}							0.05					
Task_τ_{00}							0.11					
Word_τ_{00}							0.64					
Task:Word_τ_{00}							1.50					

[a] There are no results for the model in the first subcorpus since it did not converge, but the table is kept in the same format as for the other models to facilitate comparisons.

REFERENCES

Alexopoulou, T., Michel, M., Murakami, A., & Meurers, D. (2017). Task effects on linguistic complexity and accuracy: A large-scale learner corpus analysis employing natural language processing techniques. *Language Learning*, *67*(S1), 180–208. https://doi.org/10.1111/lang.12232

Allen, B., & Becker, M. (2015). Learning alternations from surface forms with sublexical phonology. In *Lingbuzz*. http://ling.auf.net/lingbuzz/002503

Baayen, R. H., Wurm, L. H., & Aycock, J. (2007). Lexical dynamics for low-frequency complex words. *The Mental Lexicon*, *2*(3), 419–463. https://doi.org/10.1075/ml.2.3.06baa

Bakker, D., Brown, C. H., Brown, P., Egorov, D., Grant, A., Holman, E. W., Mailhammer, R., Müller, A., Velupillai, V., & Wichmann, S. (2009). Adding typology to lexicostatistics: A combined approach to language classification. *Linguistic Typology*, *13*(1), 169–181. https://doi.org/10.1515/LITY.2009.009

Beijering, K., Gooskens, C., & Heeringa, W. (2008). Predicting intelligibility and perceived linguistic distance by means of the Levenshtein algorithm. In M. van Koppen & B. Botma (Eds.), *Linguistics in the Netherlands* (pp. 13–24). John Benjamins. https://doi.org/10.1075/avt.25.05bei

Blom, E., Boerma, T., Bosma, E., Cornips, L., van den Heuij, K., & Timmermeister, M. (2020). Cross-language distance influences receptive vocabulary outcomes of bilingual children. *First Language*, *40*(2), 151–171. https://doi.org/10.1177/0142723719892794

Bolker, B. M. (2020). *Post-model-fitting procedures with glmmTMB models: Diagnostics, inference, and model output* (pp. 1–18). https://cran.r-project.org/web/packages/glmmTMB/vignettes/model_evaluation.pdf

Bosma, E., Blom, E., Hoekstra, E., & Versloot, A. (2019). A longitudinal study on the gradual cognate facilitation effect in bilingual children's Frisian receptive vocabulary. *International Journal of Bilingual Education and Bilingualism*, *22*(4), 371–385. https://doi.org/10.1080/13670050.2016.1254152

Brooks, M. E., Kristensen, K., Benthem, K. J. Van, Magnusson, A., Berg, C. W., Nielsen, A., Skaug, H. J., Mächler, M., & Bolker, B. M. (2017). glmmTMB balances speed and flexibility among packages for zero-inflated generalized linear mixed modeling. *The R*

Journal, *9*(2), 378–400.

Brooks, M. E., Kristensen, K., Darrigo, M. R., Rubim, P., Uriarte, M., Bruna, E., & Bolker, B. M. (2019). Statistical modeling of patterns in annual reproductive rates. *Ecology*, *100*(7), 1–7. https://doi.org/10.1002/ecy.2706

Brown, C. H., Holman, E. W., Wichmann, S., & Velupillai, V. (2008). Automated classification of the world's languages: A description of the method and preliminary results. *STUF – Language Typology and Universals*, *61*(4), 285–308. https://doi.org/10.1524/stuf.2008.0026

Bultena, S., Danielmeier, C., Bekkering, H., & Lemhöfer, K. (2020). The role of conflicting representations and uncertainty in internal error detection during L2 learning. *Language Learning*, *70*(S2), 75–103. https://doi.org/10.1111/lang.12401

Carrasco-Ortiz, H., Amengual, M., & Gries, S. T. (2021). Cross-language effects of phonological and orthographic similarity in cognate word recognition. *Linguistic Approaches to Bilingualism*, *11*(3), 389–417. https://doi.org/10.1075/lab.18095.car

Casaponsa, A., Antón, E., Pérez, A., & Duñabeitia, J. A. (2015). Foreign language comprehension achievement: Insights from the cognate facilitation effect. *Frontiers in Psychology*, *6*, 1–12. https://doi.org/10.3389/fpsyg.2015.00588

Cenoz, J., Leonet, O., & Gorter, D. (2021). Developing cognate awareness through pedagogical translanguaging. *International Journal of Bilingual Education and Bilingualism*, 1–15. https://doi.org/10.1080/13670050.2021.1961675

Chiswick, B. R., & Miller, P. W. (2005). Linguistic distance: A quantitative measure of the distance between English and other languages. *Journal of Multilingual and Multicultural Development*, *26*(1), 1–11. https://doi.org/10.1080/14790710508668395

Cop, U., Dirix, N., Van Assche, E., Drieghe, D., & Duyck, W. (2017). Reading a book in one or two languages? An eye movement study of cognate facilitation in L1 and L2 reading. *Bilingualism*, *20*(4), 747–769. https://doi.org/10.1017/S1366728916000213

Costa, A., Caramazza, A., & Sebastian-Galles, N. (2000). The cognate facilitation effect: Implications for models of lexical access. *Journal of Experimental Psychology: Learning Memory and Cognition*, *26*(5), 1283–1296. https://doi.org/10.1037/0278-7393.26.5.1283

De Wilde, V., Brysbaert, M., & Eyckmans, J. (2020). Learning English through out-of-school

exposure: How do word-related variables and proficiency influence receptive vocabulary learning? *Language Learning*, *70*(2), 349–381. https://doi.org/10.1111/lang.12380

De Wilde, V., Brysbaert, M., & Eyckmans, J. (2022). Formal versus informal L2 learning: How do individual differences and word-related variables influence French and English L2 vocabulary learning in Dutch-speaking children? *Studies in Second Language Acquisition*, *44*(1), 87–111. https://doi.org/10.1017/S0272263121000097

Dean, C. B., & Lundy, E. R. (2016). *Overdispersion*. Wiley StatsRef: Statistics Reference Online. https://doi.org/10.1002/9781118445112.stat06788.pub2

Dijkstra, T., Miwa, K., Brummelhuis, B., Sappelli, M., & Baayen, H. (2010). How cross-language similarity and task demands affect cognate recognition. *Journal of Memory and Language*, *62*(3), 284–301. https://doi.org/10.1016/j.jml.2009.12.003

Eberhard, D. M., Simons, G. F., & Fennig, C. D. (2021). *Ethnologue: Languages of the world (twenty-fourth edition)*. SIL International. https://www.ethnologue.com

Ecke, P. (2015). Parasitic vocabulary acquisition, cross-linguistic influence, and lexical retrieval in multilinguals. *Bilingualism*, *18*(2), 145–162. https://doi.org/10.1017/S1366728913000722

Eden, S. E. (2018). *Measuring phonological distance between languages*. University College London.

Fabozzi, F. J., Focardi, S. M., Rachev, S. T., & Arshanapalli, B. G. (2014). Model selection criterion: AIC and BIC. In *The basics of financial econometrics* (pp. 399–403). https://doi.org/10.1002/9781118856406.app5

Fontan, L., Ferrané, I., Farinas, J., Pinquier, J., & Aumont, X. (2016). Using phonologically weighted Levenshtein distances for the prediction of microscopic intelligibility. *Proceedings of the Annual Conference of the International Speech Communication Association (INTERSPEECH)*, 650–654. https://doi.org/10.21437/Interspeech.2016-431

Forthmann, B., & Doebler, P. (2021). Reliability of researcher capacity estimates and count data dispersion: A comparison of Poisson, negative binomial, and Conway-Maxwell-Poisson models. *Scientometrics*, *126*(4), 3337–3354. https://doi.org/10.1007/s11192-021-03864-8

Gooskens, C. (2006). Linguistic and extra-linguistic predictors of inter-Scandinavian

intelligibility. In J. van de Weijer & B. Los (Eds.), *Linguistics in the Netherlands* (Vol. 23, pp. 101–113). John Benjamins. https://doi.org/10.1075/avt.23.12goo

Gooskens, C., & Heeringa, W. (2004). Perceptive evaluation of Levenshtein dialect distance measurements using Norwegian dialect data. *Language Variation and Change*, *16*(3), 189–207. https://doi.org/10.1017/S0954394504163023

Gray, R. D., & Atkinson, Q. D. (2003). Language-tree divergence times support the Anatolian theory of Indo-European origin. *Nature*, *426*(6965), 435–439. https://doi.org/10.1029/2001gc000192

Green, J. A. (2021). Too many zeros and/or highly skewed? A tutorial on modelling health behaviour as count data with Poisson and negative binomial regression. *Health Psychology and Behavioral Medicine*, *9*(1), 436–455. https://doi.org/10.1080/21642850.2021.1920416

Greenhill, S. J. (2011). Levenshtein distances fail to identify language relationships accurately. *Computational Linguistics*, *37*(4), 689–698. https://doi.org/10.1162/COLI_a_00073

Gries, S. T. (2021). (Generalized linear) mixed-effects modeling: A learner corpus example. *Language Learning*, *71*(3), 757–798. https://doi.org/10.1111/lang.12448

Hall, K. C., Allen, B., Fry, M., Johnson, K., Lo, R., Mackie, S., & McAuliffe, M. (2017). *Phonological CorpusTools* (1.3). https://corpustools.readthedocs.io/en/latest/index.html

Hanulíková, A., Dediu, D., Fang, Z., Bašnaková, J., & Huettig, F. (2012). Individual differences in the acquisition of a complex L2 phonology: A training study. *Language Learning*, *62*(SUPPL. 2), 79–109. https://doi.org/10.1111/j.1467-9922.2012.00707.x

Harris, T., Yang, Z., & Hardin, J. W. (2012). Modeling underdispersed count data with generalized Poisson regression. *Stata Journal*, *12*(4), 736–747. https://doi.org/10.1177/1536867x1201200412

Hartig, F. (2020). *What does it mean if a DHARMa test is significant? #212*. https://github.com/florianhartig/DHARMa/issues/212

Hartig, F. (2021a). *DHARMa: Residual diagnostics for hierarchical (multi-level / mixed) regression models*. R package. https://cran.r-project.org/package=DHARMa

Hartig, F. (2021b). *DHARMa: Residual diagnostics for hierarchical (multi-level / mixed)*

regression models. http://web.archive.org/web/20210528100353/https://cran.r-project.org/web/packages/DHARMa/vignettes/DHARMa.html

Heeringa, W., & Prokić, J. (2018). Computational dialectology. In C. Boberg, J. Nerbonne, & W. Dominic (Eds.), *The handbook of dialectology* (pp. 330–347). John Wiley & Sons, Inc. https://doi.org/10.1002/9781118827628.ch19

Holman, E. W., Wichmann, S., Brown, C. H., Velupillai, V., Müller, A., & Bakker, D. (2008a). Advances in automated language classification. In A. Arppe, K. Sinnemäke, & U. Nikanne (Eds.), *Third workshop on quantitative investigations in theoretical linguistics (QITL3)* (pp. 40–43). University of Helsinki.

Holman, E. W., Wichmann, S., Brown, C. H., Velupillai, V., Müller, A., & Bakker, D. (2008b). Explorations in automated language classification. *Folia Linguistica*, *42*(3–4), 331–353.

Hoshino, N., & Kroll, J. F. (2008). Cognate effects in picture naming: Does cross-language activation survive a change of script? *Cognition*, *106*(1), 501–511. https://doi.org/10.1016/j.cognition.2007.02.001

Hox, J. J., Moerbeek, M., & Schoot, R. van de. (2018). *Multilevel analysis: Techniques and applications*. Routledge. https://doi.org/10.1198/jasa.2003.s281

Jarvis, S., & Pavlenko, A. (2008). *Crosslinguistic influence in language and cognition*. Routledge.

Kellerman, E. (1983). Now you see it, now you don’t. In S. Gass & L. Selinker (Eds.), *Language transfer in language learning* (pp. 112–134). Newbury House.

Kessler, B. (1995). Computational dialectology in Irish Gaelic. *Proceedings of the Seventh Conference of the European Chapter of the Association for Computational Linguistics*, 60–66. https://aclanthology.org/E95-1009

Kondrak, G. (2000). A New Algorithm for the Alignment of Phonetic Sequences. *Proceedings of the 1st North American Chapter of the Association for Computational Linguistics Conference (NAACL 2000)*, 288–295. http://dl.acm.org/citation.cfm?id=974343

Kuha, J. (2004). AIC and BIC: Comparisons of assumptions and performance. *Sociological Methods and Research*, *33*(2), 188–229. https://doi.org/10.1177/0049124103262065

Levshina, N. (2018). Probabilistic grammar and constructional predictability: Bayesian generalized additive models of help + (to) Infinitive in varieties of web-based English. *Glossa: A Journal of General Linguistics*, *3*(1), 1–22. https://doi.org/10.5334/gjgl.294

Little, C. C. (2018). *Abydos NLP/IR library for Python* (0.3.5). http://doi.org/10.5281/zenodo.1463204

Llach, M. P. A. (2010). An overview of variables affecting lexical transfer in writing: A review study. *International Journal of Linguistics*, *2*(1), E2. https://doi.org/10.5296/ijl.v2i1.445

Lüdecke, D., Ben-shachar, M. S., Patil, I., Waggoner, P., & Makowski, D. (2021). performance: An R Package for assessment, comparison and testing of statistical models. *The Journal of Open Source Software, 6*(60), Article 3139. https://doi.org/10.21105/joss.03139

Lynch, H. J., Thorson, J. T., & Shelton, A. O. (2014). Dealing with under- and over-dispersed count data in life history, spatial, and community ecology. *Ecology*, *95*(11), 3173–3180.

Makowski, D., & Lüdecke, D. (2019). *The report package for R: Ensuring the use of best practices for results reporting*. R package. https://github.com/easystats/report

Manurung, R., Ritchie, G., Pain, H., Waller, A., Black, R., & O’Mara, D. (2008). Adding phonetic similarity data to a lexical database. *Language Resources and Evaluation*, *42*(3), 319–324. https://doi.org/10.1007/s10579-008-9069-5

Marecka, M., Szewczyk, J., Otwinowska, A., Durlik, J., Foryś-Nogala, M., Kutyłowska, K., & Wodniecka, Z. (2021). False friends or real friends? False cognates show advantage in word form learning. *Cognition, 206*, 104477. https://doi.org/10.1016/j.cognition.2020.104477

Matuschek, H., Kliegl, R., Vasishth, S., Baayen, H., & Bates, D. (2017). Balancing Type I error and power in linear mixed models. *Journal of Memory and Language*, *94,* 305–315. https://doi.org/10.1016/j.jml.2017.01.001

McCallum, J. (2019). *autocorrect* (0.4.4). Python library. https://github.com/phatpiglet/autocorrect/

McCoy, R. T., & Frank, R. (2018). Phonologically Informed Edit Distance Algorithms for Word Alignment with Low-Resource Languages. *Proceedings of the Society for Computation in Linguistics (SCiL) 2018*, *1998*, 102–112.

https://doi.org/10.7275/R5251GC0

McKinney, W. (2010). Data structures for statistical computing in Python. *Proceedings of the 9th Python in Science Conference*, 51–56. http://conference.scipy.org/proceedings/scipy2010/mckinney.html

Morrissey, M. B., & Ruxton, G. D. (2018). Multiple regression is not multiple regressions: The meaning of multiple regression and the non-problem of collinearity. *Philosophy, Theory, and Practice in Biology*, *10*(3), 1–24. https://doi.org/10.3998/ptpbio.16039257.0010.003

Mortensen, D. R. (2015). *PanPhon*. https://github.com/dmort27/panphon

Mortensen, D. R., Littell, P., Bharadwaj, A., Goyal, K., Dyer, C., & Levin, L. (2016). PanPhon: A resource for mapping IPA segments to articulatory feature vectors. *Proceedings of COLING 2016, the 26th International Conference on Computational Linguistics: Technical Papers*, 3475–3484.

Nerbonne, J., & Heeringa, W. (1997). Measuring dialect distance phonetically. In J. Coleman (Ed.), *Workshop on Computational Phonology, Special Interest Group of the Association for Computational Linguistics* (Issue 1995, pp. 11–18).

Nerbonne, J., & Heeringa, W. (2001). Computational comparison and classification of dialects. *Dialectologia et Geolinguistica*, *9*, 69–84. https://doi.org/10.1515/dig.2001.2001.9.69

O'Brien, R. M. (2007). A caution regarding rules of thumb for variance inflation factors. *Quality and Quantity*, *41*(5), 673–690. https://doi.org/10.1007/s11135-006-9018-6

Oliphant, T. E. (2006). *A guide to NumPy*. Trelgol Publishing.

Otwinowska, A., Foryś-Nogala, M., Kobosko, W., & Szewczyk, J. (2020). Learning orthographic cognates and non-cognates in the classroom: Does awareness of cross-linguistic similarity matter. *Language Learning*, *70*(3), 685–731. https://doi.org/10.1111/lang.12390

Otwinowska, A., & Szewczyk, J. M. (2019). The more similar the better? Factors in learning cognates, false cognates and non-cognate words. *International Journal of Bilingual Education and Bilingualism*, *22*(8), 974–991. https://doi.org/10.1080/13670050.2017.1325834

Petroni, F., & Serva, M. (2010). Measures of lexical distance between languages. *Physica A: Statistical Mechanics and Its Applications*, *389*(11), 2280–2283. https://doi.org/10.1016/j.physa.2010.02.004

Pompei, S., Loreto, V., & Tria, F. (2011). On the accuracy of language trees. *PLoS ONE*, *6*(6), e20109. https://doi.org/10.1371/journal.pone.0020109

Poort, E. D., & Rodd, J. M. (2017). The cognate facilitation effect in bilingual lexical decision is influenced by stimulus list composition. *Acta Psychologica*, *180*, 52–63. https://doi.org/10.1016/j.actpsy.2017.08.008

R Core Team. (2021). *R: A language and environment for statistical computing*. R Foundation for Statistical Computing. https://www.r-project.org/

Rabinovich, E., Tsvetkov, Y., & Wintner, S. (2018). Native language cognate effects on second language lexical choice. *Transactions of the Association for Computational Linguistics*, *6*, 329–342. https://doi.org/10.1162/tacl_a_00024

Ringbom, H. (2007). *Cross-linguistic similarity in foreign language learning*. Multilingual Matters.

Sadat, J., Pureza, R., & Alario, F. X. (2016). Traces of an early learned second language in discontinued bilingualism. *Language Learning*, *66*(Suppl. 2), 210–233. https://doi.org/10.1111/lang.12199

Sanders, N. C., & Chin, S. B. (2009). Phonological distance measures. *Journal of Quantitative Linguistics*, *16*(1), 96–114. https://doi.org/10.1080/09296170802514138

Schepens, J., Dijkstra, T., & Grootjen, F. (2012). Distributions of cognates in Europe as based on Levenshtein distance. *Bilingualism*, *15*(1), 157–166. https://doi.org/10.1017/S1366728910000623

Schepens, J., Dijkstra, T., Grootjen, F., & van Heuven, W. J. B. (2013). Cross-Language Distributions of High Frequency and Phonetically Similar Cognates. *PLoS ONE*, *8*(5). https://doi.org/10.1371/journal.pone.0063006

Schepens, J., van der Slik, F., & van Hout, R. (2016). L1 and L2 distance effects in learning L3 Dutch. *Language Learning*, *66*(1), 224–256. https://doi.org/10.1111/lang.12150

Schepens, J., van der Slik, F., & van Houta, R. (2013a). Learning complex features: A morphological account of L2 learnability. *Language Dynamics and Change*, *3*(2), 218–

244. https://doi.org/10.1163/22105832-13030203

Schepens, J., van der Slik, F., & van Houta, R. (2013b). The effect of linguistic distance across Indo-European mother tongues on learning Dutch as a second language. In L. Borin & A. Saxena (Eds.), *Approaches to measuring linguistic differences* (pp. 199–230). De Gruyter.

Sedgwick, P. (2010). Incidence rate ratio. *BMJ*, *341*, c4804. https://doi.org/10.1136/bmj.c4804

Sellers, K. F., & Morris, D. S. (2017). Underdispersion models: Models that are "under the radar." *Communications in Statistics - Theory and Methods*, *46*(24), 12075–12086. https://doi.org/10.1080/03610926.2017.1291976

Serva, M., & Petroni, F. (2008). Indo-European languages tree by Levenshtein distance. *EPL (Europhysics Letters)*, *81*(6), 1–5. https://doi.org/10.1209/0295-5075/81/68005

Sheng, L., Lam, B. P. W., Cruz, D., & Fulton, A. (2016). A robust demonstration of the cognate facilitation effect in first-language and second-language naming. *Journal of Experimental Child Psychology*, *141*, 229–238. https://doi.org/10.1016/j.jecp.2015.09.007

Silveira, A. P. da, & Leussen, J.-W. van. (2015). Generating a bilingual lexical corpus using interlanguage normalized Levenshtein distances. *Proceedings of the 18th International Congress of Phonetic Sciences (ICPhS 2015)*, 1–5.

Sofroniev, P. (2018). *asjp* (0.0.2). Python library. https://github.com/pavelsof/asjp

Speer, R. (2020). *wordfreq 2.2.2*. PyPi. https://pypi.org/project/wordfreq/

van de Ven, M., Segers, E., & Verhoeven, L. (2019). Enhanced second language vocabulary learning through phonological specificity training in adolescents. *Language Learning*, *69*(1), 222–250. https://doi.org/10.1111/lang.12330

van der Slik, F. W. P. (2010). Acquisition of Dutch as a second language: The explanative power of cognate and genetic linguistic distance measures for 11 West European first languages. *Studies in Second Language Acquisition*, *32*(3), 401–432. https://doi.org/10.1017/S0272263110000021

Vandenberghe, B., Perez, M. M., Reynvoet, B., & Desmet, P. (2021). Combining explicit and sensitive indices for measuring L2 vocabulary learning through contextualized input and

word-focused instruction. *Studies in Second Language Acquisition*, *43*(5), 1009–1039. https://doi.org/10.1017/S0272263120000431

Vanlangendonck, F., Peeters, D., Rueschemeyer, S. A., & Dijkstra, T. (2020). Mixing the stimulus list in bilingual lexical decision turns cognate facilitation effects into mirrored inhibition effects. *Bilingualism*, *23*(4), 836–844. https://doi.org/10.1017/S1366728919000531

Virtanen, P., Gommers, R., Oliphant, T. E., Haberland, M., Reddy, T., Cournapeau, D., Burovski, E., Peterson, P., Weckesser, W., Bright, J., van der Walt, S. J., Brett, M., Wilson, J., Millman, K. J., Mayorov, N., Nelson, A. R. J., Jones, E., Kern, R., Larson, E., … Contributors, S. 1. 0. (2019). SciPy 1.0—Fundamental algorithms for scientific computing in Python. *ArXiv E-Prints*, 1–22. http://arxiv.org/abs/1907.10121

Walt, S. van der, Colbert, S. C., & Varoquaux, G. (2011). The NumPy array: A structure for efficient numerical computation. *Computing in Science & Engineering*, *13*, 22–30. https://doi.org/10.1109/MCSE.2011.37

Wichmann, S., Holman, E. W., Bakker, D., & Brown, C. H. (2010). Evaluating linguistic distance measures. *Physica A: Statistical Mechanics and Its Applications*, *389*(17), 3632–3639. https://doi.org/10.1016/j.physa.2010.05.011

Wieling, M., Bloem, J., Mignella, K., Timmermeister, M., & Nerbonne, J. (2014). Measuring foreign accent strength in English: Validating Levenshtein distance as a measure. *Language Dynamics and Change*, *4*(2), 253–269.

Wieling, M., Heeringa, W., & Nerbonne, J. (2007). An Aggregate Analysis of Pronunciation in the Goeman-Taeldeman-Van Reenen-Project Data. *Taal En Tongval*, *59*, 84–116.

Wieling, M., Nerbonne, J., Bloem, J., Gooskens, C., Heeringa, W., & Baayen, R. H. (2014). A cognitively grounded measure of pronunciation distance. *PLoS ONE*, *9*(1), e75734. https://doi.org/10.1371/journal.pone.0075734

Winter, B. (2019). *Statistics for linguists: An introduction using R*. Routledge. https://doi.org/10.4324/9781315165547

Xia, C. M. (2017). Psychotypology of Chinese learners of English and its influence on the acquisition of metaphorical expressions: An offline study. *Cambridge Occasional Papers in Linguistics*, *10*, 237–255.

Zhang, J., Wu, C., Zhou, T., & Meng, Y. (2019). Cognate facilitation priming effect is

modulated by writing system: Evidence from Chinese-English bilinguals. *International Journal of Bilingualism*, *23*(2), 553–566. https://doi.org/10.1177/1367006917749062

Zhang, L. (2018). *A More Sensitive Edit-Distance for Measuring Pronunciation Distances and Detecting Loanwords*. University of Groningen.

Zhu, F. (2012). Modeling overdispersed or underdispersed count data with generalized Poisson integer-valued GARCH models. *Journal of Mathematical Analysis and Applications*, *389*(1), 58–71. https://doi.org/10.1016/j.jmaa.2011.11.042

Zhu, Y., & Mok, P. P. K. (2020). Visual recognition of cognates and interlingual homographs in two non-native languages. *Linguistic Approaches to Bilingualism*, *10*(4), 441–470. https://doi.org/10.1075/lab.17049.zhu
